# Implications of the Propagation Method for the Phytochemistry of *Nepeta cataria* L. throughout a Growing Season

**DOI:** 10.3390/molecules29092001

**Published:** 2024-04-26

**Authors:** Erik Nunes Gomes, Bo Yuan, Harna K. Patel, Anthony Lockhart, Christian A. Wyenandt, Qingli Wu, James E. Simon

**Affiliations:** 1 New Use Agriculture and Natural Plant Products Program, Department of Plant Biology, Rutgers University, New Brunswick, NJ 08901, USA; 2 Federal Agency for Support and Evaluation of Graduate Education (CAPES), Ministry of Education of Brazil, Brasilia 70040-020, DF, Brazil; 3 Rutgers Core Facility for Natural Products and Bioanalysis, Rutgers University, New Brunswick, NJ 08901, USA; 4 Department of Medicinal Chemistry, Ernest Mario School of Pharmacy, Rutgers University, Piscataway, NJ 08854, USA; 5 New Jersey Agricultural Experiment Station, Rutgers Agricultural Research and Extension Center (RAREC), Department of Plant Biology, Rutgers University, Bridgeton, NJ 08302, USA

**Keywords:** catnip, flowering, iridoid terpenes, nepetalactone, ontogeny, phenolic compounds

## Abstract

Catnip (*Nepeta cataria* L.) plants produce a wide array of specialized metabolites with multiple applications for human health. The productivity of such metabolites, including nepetalactones, and natural insect repellents is influenced by the conditions under which the plants are cultivated. In this study, we assessed how field-grown catnip plants, transplanted after being propagated via either single-node stem cuttings or seeds, varied regarding their phytochemical composition throughout a growing season in two distinct environmental conditions (Pittstown and Upper Deerfield) in the state of New Jersey, United States. Iridoid terpenes were quantified in plant tissues via ultra-high-performance liquid chromatography with triple quadrupole mass spectrometry (UHPLC-QqQ-MS), and phenolic compounds (phenolic acids and flavonoids) were analyzed via UHPLC with diode-array detection (UHPLC-DAD). The highest contents of total nepetalactones in Pittstown were found at 6 weeks after transplanting (WAT) for both seedlings and cuttings (1305.4 and 1223.3 mg/100 g, respectively), while in Upper Deerfield, the highest contents for both propagules were at 11 WAT (1247.7 and 997.1 mg/100 g, respectively) for seed-propagated and stem cuttings). The highest concentration of nepetalactones was associated with floral-bud to partial-flowering stages. Because plants in Pittstown accumulated considerably more biomass than plants grown in Upper Deerfield, the difference in nepetalactone production per plant was striking, with peak productivity reaching only 598.9 mg per plant in Upper Deerfield and 1833.1 mg per plant in Pittstown. Phenolic acids accumulated in higher contents towards the end of the season in both locations, after a period of low precipitation, and flavone glycosides had similar accumulation patterns to nepetalactones. In both locations, rooted stem cuttings reached their maximum nepetalactone productivity, on average, four weeks later than seed-propagated plants, suggesting that seedlings have, overall, better agronomic performance.

## 1. Introduction

The Lamiaceae family comprises more than 7000 species and is one of the most studied plant groups regarding its ethnopharmacology and the biosynthesis of secondary metabolites of scientific and economic interest [1]. Lamiaceae is the family with the highest number of aromatic species, producing volatile metabolites of commercial importance due to their use in the fragrance and flavor industries [2]. Within Lamiaceae, plants from the genus *Nepeta*, and most notably the species *N. cataria* L. (catnip), are known to produce volatile compounds with attractant effects on domestic cats and other felid species and are widely used in the pet toy industry [3,4].

In addition to the characteristic effects on cats that gave catnip its common name, natural products from this plant are widely recognized as arthropod repellents [5]. *N. cataria* products have been reported to repel mosquitoes, ticks, bed bugs, and other important arthropod threats to human and animal health [6,7,8], which increased the interest in the cultivation of this species as a crop [3]. Different extracts from *N. cataria* have also shown numerous biological activities in vivo and in vitro, including antioxidant, antibacterial, antifungal, antiviral, anti-convulsant, myorelaxant, antidiabetic, anti-inflammatory, analgesic, anti-depressant, anthelmintic, nematicidal, trypanicidal, hepatoprotective, and immunomodulatory activities, among others [3,9].

The arthropod-repellent and cat-stimulant effects are attributed to volatile iridoid terpenes, synthetized by *Nepeta* species, most notably different isomers of the monoterpene nepetalactone [4,5,6]. *N. cataria* can also produce other iridoid terpenes such as dihydronepetalactone, nepetalactam and nepetalic acid [10], which, although not as extensively studied as nepetalactones, have also demonstrated potential to be used as arthropod repellents or precursors of repellent molecules [11,12,13]. Besides volatile terpenes, catnip also produces a wide array of phenolic compounds, including phenolic acids such as caffeic and rosmarinic acids and flavones such as apigenin and luteolin, as well as their glycosides [14,15]. These phenolic compounds have been reported to affect arthropod behavior [16,17,18] and have the potential to be included in integrated pest management systems and other applications related to human health.

Given the abundant production of bioactive natural products, particularly those related to arthropod repellency, interest in growing catnip as a crop has increased, and research has focused on the identification and development of new genetic materials with stable phytochemical characteristics suited for cultivation conditions in different regions, including improved plant architecture for mechanical harvesting [10,19,20,21].

Along with genotype, environmental conditions can also significantly impact the production of plant secondary metabolites in plants [22]. In the case of cultivated species, environmental interactions are often determined by crop management. Cultivation conditions such as propagation methods, plant densities, fertilization, irrigation, harvesting times (seasonality and daily variation), and post-harvesting procedures, among others, are known to affect the productivity of plant natural products both quantitively and qualitatively [23,24,25,26,27].

For *N. cataria*, the effect of seasonality on the productivity of secondary metabolites has been reported. Weekly harvests from a wild catnip population showed a significant variation in the *Z*,*E*/*E*,*Z* nepetalactone (cis, trans/trans, cis) ratio, with a higher ratio during the first harvests in May and a significant decline in subsequent harvests up to July, possibly associated with the plant’s developmental stages and ecological interactions [28]. Additionally, a series of studies have been conducted showing that the chemical composition of cultivated catnip is strongly influenced by the plant’s developmental stage [29,30,31].

Regarding propagation methods, for commercial cultivation, catnip is mainly propagated via seeds, either by direct sowing or producing seedlings under protected environments and then transplanting to field conditions [3]. However, the vegetative propagation of the species via stem cuttings is possible and has been previously described [32,33,34]. Previous studies on catnip propagation have concluded that transplanting seedlings produces increments in yields that are enough to make it more profitable than direct sowing [35] and that terminal stem cuttings transplanted after 2 or 3 weeks of rooting have more biomass allocated to shoots than terminal cuttings propagated for longer periods [33]. However, to date, there are no reports addressing the direct comparison between transplanting seedlings and rooted stem cuttings in terms of the biomass accumulation and phytochemistry of *N. cataria*.

Although the clonal propagation of cultivated plant species offers the advantage of the lower variation of agronomically valuable genotypes [36], there are fundamental morphological and physiological differences between rooted cuttings and seedlings. Such differences are well documented for tree species and are mainly related to root architecture and plant juvenility [37,38,39,40], which have the potential to alter the mechanisms by which those plants interact with the environment as well as their developmental patterns.

Despite being commonly studied in fruit crops and forest trees, the implications of propagation methods for the development, physiology, and productivity of herbaceous aromatic plants have not been extensively researched. We hypothesize that rooted stem cuttings and self-pollinated seedlings of *N. cataria* will have distinct responses to seasonality and environmental interactions, which can lead to significant differences in the accumulation of biomass and secondary metabolites of interest. Therefore, the aim of this study was to assess the accumulation of biomass, iridoid terpenes, phenolic acids, and flavones and their glycosides in *N. cataria* plants propagated either by stem cuttings or seeds throughout a growing season in two experimental locations, as well as to discuss the implications of such differences in terms of ecophysiological interactions and crop productivity.

## 2. Results

The ANOVA tables for all the assessed variables in each of the experimental sites, as well as the complete set of pairwise comparisons, including the interaction effects between propagule types and times of harvests, are presented in Supplementary Tables S1–S12. In the following sections, we present the main interactions and the accumulation patterns of secondary metabolites in catnip plants according to types of propagules and harvest time.

### 2.1. Nepetalactone Concentrations

#### 2.1.1. Pittstown

**Figure 1:**
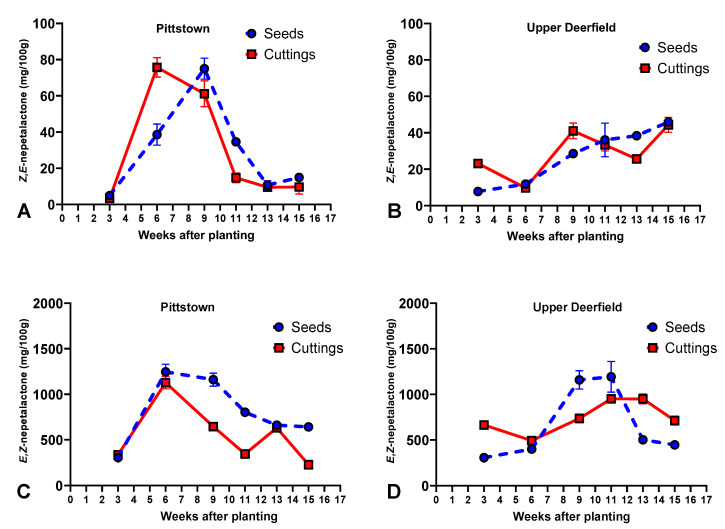
Average contents (mg per 100 g of dry biomass) of *E*,*Z*-nepetalactone, *Z*,*E*-nepetalactone, and total nepetalactones in aerial parts of catnip (*Nepeta cataria* L.) propagated by transplanting seedlings (Seeds) and rooted stem cuttings (Cuttings) and harvested at different times in experimental fields in Pittstown (**A**,**C**) and Upper Deerfield (**B**,**D**), state of New Jersey, United States. Bars represent the standard deviation of the mean. Pittstown Tukey’s HSD (*p* ≤ 0.05) for *E*,*Z*-nepetalactone: 59.9 for propagule types and 102.3 for harvest time; *Z*,*E*-nepetalactone: 6.2 for propagule types and 10.2 for harvest time. Upper Deerfield Tukey’s HSD (*p* ≤ 0.05) for *E*,*Z*-nepetalactone: 86.4 for propagule types and 139.2 for harvest time; *Z*,*E*-nepetalactone: 5.6 for propagule types and 8.4 for harvest time.

*Z*,*E*-nepetalactone concentrations were higher in stem cuttings at 9, 11, and 15 weeks after planting (WAT) and equivalent in weeks 3 and 13, and stem cuttings had a higher concentration than seedlings when harvested at 6 WAT. The highest concentration of *Z*,*E*-nepetalactone in seedlings occurred at 9 weeks after planting (75.1 mg/100 g), while for stem cuttings, it occurred at 6 WAT (75.7 mg/100 g). In both propagule types, there was a continuous reduction of *Z*,*E*-nepetalactone content after the peak production up until week 13, when there was a similar concentration to that of plants harvested 15 WAT (Figure 1A, Supplementary Table S3).

*E*,*Z*-nepetalactone concentrations were higher in plants propagated via seeds than in stem cuttings in all harvest times, except for at 3 and 13 WAT, when there was no statistical difference between the propagule types. The highest *E*,*Z*-nepetalactone concentrations in seedlings were found at 6 and 9 WAP (1245.7 and 1160.6 mg/100 g of dry weight, respectively, not statistically different from each other), while for stem cuttings, the peak concentration happened at 6 WAT only, followed by a marked decline until week 11, a significant increase in week 13, and another decrease at 15 WAT. For seedlings, after week 9, the concentrations of *E*,*Z*-nepetalactone decreased until 13 WAT and remained at the same level in week 15 (Figure 1C, Supplementary Table S3). Total nepetalactone concentrations showed the same patterns of accumulation of *E*,*Z*-nepetalactone, reaching the peak of 1305.4 mg/100 g in plants propagated by seeds and 1223.3 mg/100 g in stem cuttings (Supplementary Table S3).

#### 2.1.2. Upper Deerfield

For *Z*,*E*-nepetalactone, there were no statistical differences between stem cuttings and seedlings at 6, 11, and 15 WAT; seedlings had higher concentrations than cuttings when harvested at 13 WAT; and stem cuttings had higher concentrations than seedlings at 3 and 9 WAT. Plants propagated via seeds had a linear increase in *Z*,*E*-nepetalactone concentrations throughout the growing season, reaching the highest concentration (45.9 mg/100 g) at 15 WAT. Stem cuttings had two peak concentrations of *Z*,*E*-nepetalactones: the first at week 9 (41 mg/100 g) and the second at 15 WAT (44.3 mg/100 g) (Figure 1B, Supplementary Table S4).

Plants propagated by seeds reached higher concentrations of *E*,*Z*-nepetalactones than stem cuttings when harvested at 9 and 11 WAT, while stem cuttings had higher concentrations than seedlings when harvested at 3, 6, 13, and 15 WAT. Seedlings reached their highest concentrations of *E*,*Z*-nepetalactones at weeks 9 and 11 (1157.9 and 1192 mg/100 g, respectively, not statistically different from each other), followed by a steep decline at week 13 and maintaining similar levels at 15 WAP. Stem cuttings had an *E*,*Z*-nepetalactone concentration of 663.8 mg/100 g when harvested at 3 WAT, followed by a reduction in week 6, and increases up to weeks 11 and 13, when they reached their highest concentrations (950.7 and 949.5 mg/100 g, respectively, not statistically different from each other) (Figure 1D, Supplementary Table S4). Total nepetalactones had the same accumulation patterns as *E*,*Z*-nepetalactone, with peaks of 1247.7 and 997.1 mg/100 g for seedlings and stem cuttings, respectively (Supplementary Table S4).

### 2.2. Biomass Accumulation and Total Nepetalactone Yield

#### 2.2.1. Pittstown

**Figure 2:**
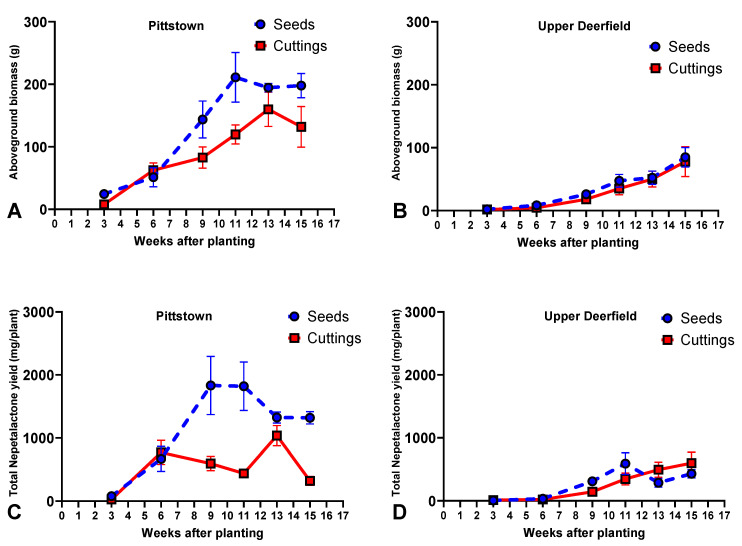
Average biomass (g per plant) and total nepetalactone yield (mg per plant) in aerial parts of catnip (*Nepeta cataria* L.) propagated by transplanting seedlings (Seeds) and rooted stem cuttings (Cuttings) and harvested at different times in experimental fields in Pittstown (**A**,**C**) and Upper Deerfield (**B**,**D**), state of New Jersey, United States. Bars represent the standard deviation of the mean. Pittstown Tukey’s HSD (*p* ≤ 0.05) for biomass: 39.1 for propagule types and 55.4 for harvest time; Total nepetalactone yield: 347.7 for propagule type and 480.8 for harvest time. Upper Deerfield Tukey’s HSD (*p* ≤ 0.05) for biomass: 20.5 for propagule types and 26.3 for harvest time; Total nepetalactone yield: 165.4 for propagule type and 215.2 for harvest time.

Seedlings accumulated more biomass and had a higher nepetalactone yield than stem cuttings at 9, 11, and 15 WAT, and there were no statistically significant differences between propagule types at 3, 6, and 13 WAT. For biomass accumulation, seedlings and stem cuttings had similar patterns across time, with linear increases from week 3 to week 11 and similar levels in weeks 13 and 15 (Figure 2A, Supplementary Table S3). For nepetalactone yield, seedlings exhibited linear increases from week 3, reaching peak productivity in weeks 9 and 11 (1833.1 and 1821.0 mg per plant, respectively, not statistically different from each other), with lower values at 13 and 15 WAT. For stem cuttings, plants harvested at week 3 had a relatively low yield (26.9 mg per plant); there was a significant increase at 6 weeks after planting (770.4 mg per plant); and they reached up to 1036.5 mg per plant at 13 weeks after planting, with a reduction to 320.4 mg per plant in week 15 (Figure 2C, Supplementary Table S3).

#### 2.2.2. Upper Deerfield

In Upper Deerfield, the interaction effect between harvest times and propagules was not statistically significant for the accumulation of biomass, and only the main effect of harvest time affected this variable (*p* ≤ 0.01). The pattern of biomass accumulation was a linear increase across the growing season, reaching the highest biomass (average of 81.24 g per plant) 15 weeks after planting (Figure 2B, Supplementary Table S4). As for the nepetalactone yield, there were no statistically significant differences between seedlings and stem cuttings at 3 and 6 WAT. Seedlings outperformed stem cuttings at 9 and 11 WAT, and stem cuttings outperformed seedlings at 13 and 15 WAT. While stem cuttings had a linear increase in nepetalactone yield across the season (598.9 mg per plant at 15 WAT), seedlings reached a peak at 11 WAT (591.7 mg per plant) (Figure 2D, Supplementary Table S4).

### 2.3. Nepetalic Acid, Dihydronepetalactone and Nepetalactam Concentrations

#### 2.3.1. Pittstown

**Figure 3:**
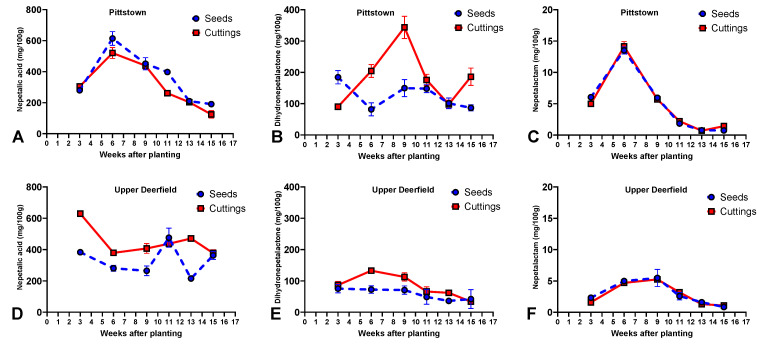
Average contents (mg per 100 g of dry biomass) of nepetalic acid, dihydronepetalactone and nepetalactam in aerial parts of catnip (*Nepeta cataria* L.) propagated by transplanting seedlings (Seeds) and rooted stem cuttings (Cuttings) and harvested at different times in experimental fields in Pittstown (**A**–**C**) and Upper Deerfield (**D**–**F**), state of New Jersey, United States. Bars represent the standard deviation of the mean. Pittstown Tukey’s HSD (*p* ≤ 0.05) for nepetalic acid: 28.7 for propagule types and 47.1 for harvest time; dihydronepetalactone: 13.0 for propagule types and 18.6 for harvest time; nepetalactam: 0.5 for propagule type and 0.8 for harvest time. Upper Deerfield Tukey’s HSD (*p* ≤ 0.05) for nepetalic acid: 33.3 for propagule types and 49.8 for harvest time; dihydronepetalactone: 12.8 for propagule types and 10.6 for harvest time; nepetalactam: 0.3 for propagule type and 0.4 for harvest time.

Nepetalic acid concentrations were higher in seedlings than in stem cuttings at 6, 11, and 15 WAT. At 3, 9, and 13 WAT, there were no significant differences between propagule types for this metabolite. Both stem cuttings and seedlings reached the highest concentration of nepetalic acid (614.3 and 520.5 mg/100 g, respectively) at 6 WAT, followed by a linear decrease until 15 WAT (Figure 3A, Supplementary Table S3).

Regarding dihydronepetalactone, seed-propagated plants had higher concentrations than stem cuttings at 3 WAT. At 6, 9, 11, and 15 WAT, stem cuttings were superior to seed-propagated plants regarding concentrations of this metabolite. For stem cuttings, there was a linear increase in dihydronepetalactone concentrations up to week 9, when the plants reached the peak concentration (343.8 mg/100 g), followed by lower concentrations in weeks 11 and 13 and a new increase at 15 WAT. For seed-propagated plants, a different pattern was observed, with the highest content being registered at 3 WAT (184.4 mg/100 g), a reduction at 6 WAT, a slight increase in weeks 9 and 11, followed by a decrease in weeks 13 and 15 (Figure 3B, Supplementary Table S3).

For nepetalactam concentrations, seedlings had statistically significantly higher values than stem cuttings at 3 WAT. Stem cuttings outperformed seedlings at 6 and 15 WAT, and there were no statistical differences between propagule types at 9, 11, and 15 WAT for this metabolite. The pattern of nepetalactam accumulation of both stem cuttings and seedlings across the growing season was similar to that of nepetalic acid, with plants reaching the highest concentrations at 6 WAT (13.5 and 14.1 mg/100 g for seedlings and stem cuttings, respectively), followed by a linear decrease up until week 15 (Figure 3C, Supplementary Table S3).

#### 2.3.2. Upper Deerfield

Stem cuttings had higher nepetalic acid concentrations than seed-propagated plants at 3, 6, 9, and 13 WAT, while seed-propagated plants had the highest concentration at 11 WAT. At 15 WAT, there were no statistical differences between propagule types. Stem cuttings had the highest concentration of nepetalic acid at 3 WAT (629.8 mg/100 g), followed by a reduction at 6 WAT, a linear increase up to week 13, and a new reduction at 15 WAT. For seedlings, there was a clear peak at 11 WAT (475.9 mg/100 g), with intermediate values at 3 and 15 WAT and the lowest contents at 9 and 13 WAT (Figure 3D, Supplementary Table S4).

Plants propagated by stem cuttings had higher dihydronepetalactone concentrations than seedlings at all harvest times, except for 3 and 15 WAT when there were no statistical differences between propagule types. Stem cuttings reached a peak of dihydronepetalactone concentration at 6 WAT (132.6 mg/100 g), followed by decreases until week 15. Seed-propagated plants reached the highest concentrations at 3, 6, and 9 WAT (75.3, 72.3, and 70.7, respectively, not statistically different from each other), followed by lower contents in weeks 11, 13, and 15 (Figure 3E, Supplementary Table S4).

As for nepetalactam concentrations, there were no statistical differences between propagule types in different harvest times, except for 11 WAT, when stem cuttings outperformed seedlings. The overall trend of both stem cuttings and seedlings included a peak of nepetalactam concentrations at 9 WAT (5.5 and 5.2 mg/100 g, for seedlings and stem cuttings, respectively), with significant decreases in concentrations until 15 WAT (Figure 3F, Supplementary Table S4).

### 2.4. Caffeic Acid and Rosmarinic Acid

#### 2.4.1. Pittstown

**Figure 4:**
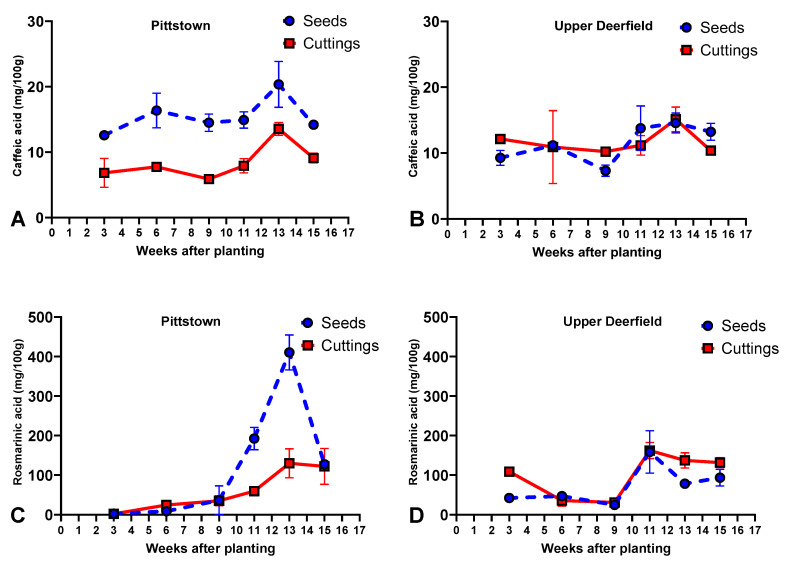
Average contents (mg per 100 g of dry biomass) of caffeic acid and rosmarinic acid in aerial parts of catnip (*Nepeta cataria* L.) propagated by transplanting seedlings (Seeds) and rooted stem cuttings (Cuttings) and harvested at different times in experimental fields in Pittstown (**A**,**C**) and Upper Deerfield (**B**,**D**), state of New Jersey, United States. Bars represent the standard deviation of the mean. Pittstown Tukey’s HSD (*p* ≤ 0.05) for caffeic acid: 2.7 for propagule types and 4.4 for harvest time; rosmarinic acid: 19.1 for propagule types and 25.5 for harvest time. Upper Deerfield Tukey’s HSD (*p* ≤ 0.05) for caffeic acid: 28.733 for propagule types and 5.7 for harvest time; rosmarinic acid: 36.0 for propagule types and 49.1 for harvest time.

In Pittstown, seed-propagated plants had a higher concentration of caffeic acid than stem cuttings. Caffeic acid concentrations at 13 WAT (average of 16.9 mg/100 g) were superior to other harvest times, which did not differ amongst themselves (Figure 4A, Supplementary Table S7).

As for rosmarinic acid, seedlings were superior to stem cuttings at 11 and 13 WAT and there were no differences between propagule types for plants harvested at 3, 6, 9, and 15 WAT. Seedlings had small increases in rosmarinic acid concentrations from week 3 to week 9 and more pronounced increases at 11 and 13 WAT when plants reached the highest concentration of this phenolic acid (410.4 mg/100 g). This was followed by a reduction at 15 WAT. Stem cuttings showed a similar pattern, though with smaller increases than seedlings at weeks 11 and 13 and without a significant reduction in rosmarinic acid levels at 15 WAT (Figure 4C, Supplementary Table S7).

#### 2.4.2. Upper Deerfield

In Upper Deerfield, there were no differences between stem cuttings and seedlings regarding caffeic acid accumulation. Overall, plants reached the highest concentrations of caffeic acid at 13 WAT (14.8 mg/100 g), although results were not statistically significant from weeks 11 and 15 (Figure 4B, Supplementary Table S8).

Stem cuttings had higher concentrations of rosmarinic acid than seedlings at 3, 13, and 15 WAT, and propagule types were not statistically different for the remaining harvest times. Seedlings showed relatively small concentrations of rosmarinic acid in the first three harvests (24.6 to 42.1 mg/100 g), reaching the highest concentration at 11 WAT (158.7 mg/100 g), followed by a reduction at 13 and 15 WAT. Stem cuttings started at 108.8 mg/100 g, followed by smaller concentrations at 6 and 9 WAT, and reached the highest mean concentration at 11 WAT (162.3 mg/100 g), which was not statistically different from results at 13 and 15 WAT (Figure 4D, Supplementary Table S8).

### 2.5. Apigenin, Luteolin, and Flavone Glycosides

#### 2.5.1. Pittstown

**Figure 5:**
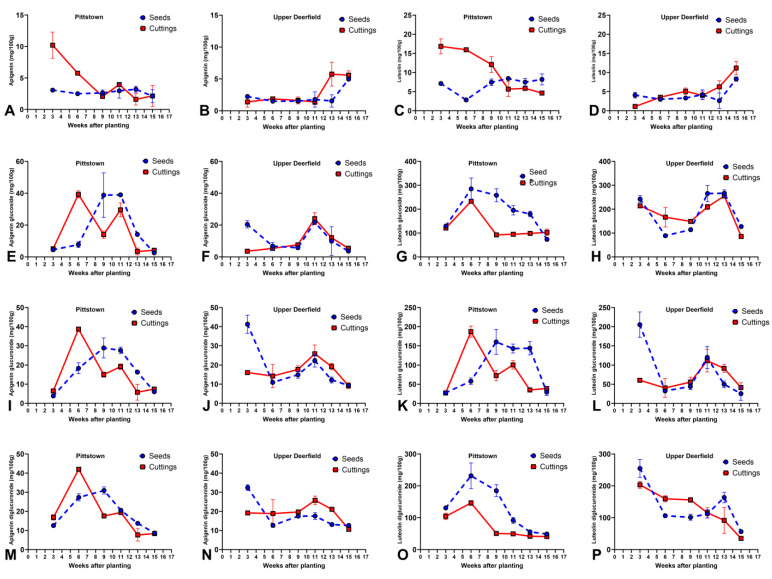
Average contents (mg per 100 g of dry biomass) of apigenin (**A**,**B**), luteolin (**C**,**D**), apigenin glucoside (**E**,**F**), luteolin glucoside (**G**,**H**), apigenin glucuronide (**I**,**J**), luteolin glucuronide (**K**,**L**), apigenin diglucuronide (**M**,**N**), and luteolin diglucuronide (**O**,**P**) in aerial parts of catnip (*Nepeta cataria* L.) propagated by transplanting seedlings (seed) and rooted stem cuttings (cutting) and harvested at different times in experimental fields in Pittstown and Upper Deerfield, state of New Jersey, United States. Bars represent the standard deviation of the mean (*n* = 3). The unfolding of the interaction effects and post hoc analyses are presented as supplementary materials (Supplementary Tables S9–S12).

In Pittstown, apigenin concentrations were higher in stem cuttings than seedlings at 3 and 6 WAT, and, for the remaining harvest times, there were no statistical differences between propagule types. Stem cuttings had their highest apigenin concentrations at 3 WAT (10.2 mg/100 g), followed by decreases at 6 and 9 WAT, and keeping similar concentrations until 15 WAT. Apigenin levels in seedlings did not vary significantly across different harvest times (Figure 5A, Supplementary Table S7).

As for luteolin, stem cuttings had higher concentrations than seed-propagated plants at 3, 6, and 9 WAT. Propagule types were not statistically different at 11 and 13 WAT, and seed-propagated plants had higher luteolin concentrations than stem cuttings at 15 WAT. Stem cuttings reached the highest luteolin concentration at 3, 6, and 9 WAT (from 16.8, 15.9, and 12.1 mg/100 g, respectively, not statistically different from each other), with a decrease at 11 WAT and maintaining low levels at 13 and 15 WAT. Seedlings reached the highest luteolin concentrations at 11 and 15 WAT (8.4 and 8.2 mg/100 g, respectively, not statistically different from each other) (Figure 5C, Supplementary Table S7).

All three apigenin glycosides had similar patterns of accumulation in Pittstown, with stem cuttings reaching their peak concentration at 6 WAT (39.3, 38.7, and 42.0 mg/100 g, respectively, for glucoside, glucuronide, and diglucuronide), followed by a decrease in the concentrations in week 9, an increase in week 11. and lower concentrations at 13 and 15 WAT. For seed-propagated plants, a nearly quadratic pattern was observed across the season, reaching peak concentrations at both 9 and 11 WAT for apigenin glucoside and apigenin glucuronide and exclusively at week 9 for apigenin diglucuronide (Figure 5E,I,M, Supplementary Table S11).

Luteolin glucoside and diglucuronide had a similar pattern of accumulation in stem cuttings and seedlings across the growing season, with both seedlings and stem cuttings reaching peak concentrations at 6 WAT, followed by a gradual reduction in seedlings until week 15 and a more pronounced reduction in stem cuttings in week 9, maintaining relatively stable contents until week 15. Overall, seed-propagated plants had a higher accumulation of luteolin glucoside and diglucuronide than stem cuttings until 13 WAT (Figure 5G,O, Supplementary Table S11). Luteolin glucuronide had a pattern similar to that of apigenin glycosides, with stem cuttings reaching peak concentrations earlier than seedlings (Figure 5K, Supplementary Table S11).

#### 2.5.2. Upper Deerfield

For both apigenin and luteolin, there were no statistical differences between propagules and only the main effect of harvest time affected the accumulation of these metabolites, with the highest concentrations being observed at the last harvest, 15 WAT (averages of 5.27 and 9.73 mg/100 g, respectively, of apigenin and luteolin) (Figure 5B,D, Supplementary Table S8).

Similar to the observed in the Pittstown site, in Upper Deerfield, all three apigenin glycosides had a comparable accumulation pattern across different harvest times. Seed-propagated plants had their highest concentration of apigenin glycosides at 3 WAT (20.4, 41.3, and 32.4 mg/100 g, respectively, for apigenin glucoside, glucuronide, and diglucuronide), followed by smaller concentrations at 6 and 9 WAT. There was a second peak at 11 WAT, followed by smaller concentrations at 13 and 15 WAT. Stem cuttings had relatively stable concentrations at 3, 6, and 9 WAT, reaching the highest concentrations at 11 WAT (24.0, 25.8, and 25.8 mg/100 g, respectively, for apigenin glucoside, glucuronide, and diglucuronide) (Figure 5F,J,N, Supplementary Table S12).

Luteolin glucuronide had a similar pattern to apigenin glycosides, with the highest concentration at 3 WAT for seed-propagated plants (205.3 mg/100 g) and at 11 WAT for stem cuttings (111.5 mg/100 g). Luteolin glucoside reached the highest concentrations for seed-propagated plants at 3, 11, and 13 WAT, while stem cuttings had peak concentrations at 3 and 13 WAT. As for luteolin diglucuronide, the two highest concentrations in stem cuttings occurred at 3 and 13 WAT, and for stem cuttings, the highest concentration was at 3 WAT, with a continuous decline until 15 WAT (Figure 5H,L,P, Supplementary Table S12). No clear differences in flavone glycosides were observed across the season between stem cuttings and seedlings, except at 3 WAT, when seedlings accumulated more of those metabolites than rooted stem cuttings.

## 3. Discussion

In this study, we describe the accumulation of biomass, iridoid terpenes, phenolic acids, flavones, and flavone glycosides throughout a growing season in *Nepeta cataria* L. plants propagated by stem cuttings or seedlings in two different experimental locations.

Medicinal and aromatic plants are known to be strongly influenced by seasonality, and dramatic changes in the contents of secondary metabolites have been reported for many commercially important species, including catnip [28,41,42]. Seasonal influence on the productivity of phytochemicals is often associated with changes in environmental patterns across time, such as changes in temperature, precipitation, and radiation [41], which can affect the transcriptional, post-transcriptional, translational, and post-translational regulations of secondary metabolite pathways [43].

Along with the direct influence on the biosynthetic pathways of phytochemicals, seasonality factors such as air temperature and solar radiation can have an indirect effect on secondary metabolite production by triggering developmental changes in plants, including the induction of flowering [44,45]. Hence, in our study, it is possible to infer that the differences in the time of flowering between catnip grown in Pittstown and Upper Deerfield were, at least to some extent, related to the effects of lower ambient temperature in Pittstown, since decreasing ambient temperatures are known to induce flowering in many plant species [46,47]. The first appearance of floral buds in Pittstown occurred when monthly average temperatures reached 22.1 °C in August, while in Upper Deerfield, the average August temperature was 24 °C, and floral buds only appeared in September when temperatures were below 22.1 °C (Table 1 and Table 2).

The influence of developmental stages (ontogeny) is extensively studied in medicinal and aromatic plants, and can, in some cases, be responsible for dramatic variations in the chemical composition of species [48,49]. The ontogenetic regulation of plant secondary metabolites is thought to be the result of evolutionary relationships with biotic and abiotic factors, such as winter temperatures and herbivory, which led the plants to chemically adapt to protect more vulnerable tissues; allocate resources to reproductive organs; or attract pollinators, among others [49,50]. In catnip, particularly, the transition to flowering seems to be associated with substantial changes in the accumulation of secondary metabolites [23,28,29,30,31,51]. In this study, the difference in the time of flowering initiation seems to be a major contributor to Pittstown plants having reached peak concentrations of the main nepetalactone isomer, *E*,*Z*-nepetalactone, earlier than plants grown in Upper Deerfield (Figure 1). Differences in flowering time between the two experimental sites were also likely related to the pattern of biomass accumulation, which reached peaks at 11 WAT and 13 WAT (for seedlings and stem cuttings, respectively) in Pittstown. Later harvests in Pittstown had reductions associated with leaf and inflorescence senescence. In Upper Deerfield, biomass increased linearly until the final harvest, when the inflorescences never fully senesced (Figure 2).

In addition to potential developmental implications of distinct environments, the differences in total nepetalactone yield between Pittstown (reaching up to 1833.1 mg per plant) and Upper Deerfield (reaching up to 598.9 mg per plant) can also be directly associated with the weather conditions of each location. At peak concentrations, the contents varied little between the two fields (averages of 1264.4 and 1122.4 mg/100 g in Pittstown and Upper Deerfield, respectively), so the differences observed in yield (Figure 2) are mostly determined by the differences in plant biomass production. A combination of milder temperatures and higher precipitation volume (Table 1) are likely to be significant contributors to the best performance of plants grown in Pittstown. Higher water availability and ideal ambient temperatures are extensively reported as improving stomatal conductance and net assimilation rates in medicinal and aromatic plants [52,53], and studies with *N. cataria* demonstrated that the biomass of the plants tends to significantly decrease under low availability of water [25,54].

In this study, overall, the highest concentrations of nepetalactones (both isomers) in plant tissues as well as related terpenes (nepetalic acid and nepetalactam) were associated with the floral-bud to partial-flowering stages. Previous research on ideal harvest stages for catnip has pointed out that flowering stages have superior yields when compared to vegetative and post-flowering phases [29,31], which is consistent with the findings of our study. Moreover, a more detailed study of the phenological stages of the species showed somewhat contrasting results, evidencing that there were higher contents of nepetalactone-rich essential oil during full flowering than in the flower-bud stage [30], which is something we did not observe in the present study. Similar results were also reported for lemon catnip (*N. cataria* var. *citriodora*), when the full-flowering phenophase was superior to the flower-bud and early-flowering stages in terms of the accumulation of an essential oil rich in nerol, citronellol, and geraniol, characteristic of this chemotype [55]. Other studies with more similar results have shown a higher accumulation of secondary metabolites, including *E*,*Z*-nepetalactone, at the beginning of flowering and in the flower-bud stage for *N. cataria* and *N. cataria* var. *citriodora* [23,28,51]. Such discrepancies can be attributed to the influence of factors such as genotypes and environmental interactions and emphasize the need to adapt agronomic practices to maximize yields in different regions.

Despite some differences in the literature regarding whether the highest essential oil and nepetalactone contents exist in the flower-bud, early-flowering, or full-flowering stage, it seems consensual that there is an increase in the production of terpenes in *N. cataria* from the moment the plant transitions from the vegetative to the reproductive stage. It also seems well established that, after the flowering stage, there is a reduction in the accumulation of iridoid terpenes. This ontogenetic pattern of secondary metabolite accumulation is relatively common for herbaceous semi-perennial plant species such as catnip. In stevia (*Stevia rebaudiana* Bert.), for example, early flowering is associated with a higher accumulation of diterpene glycosides [26], and, as flowering progresses and fruits and seeds start to develop, a reduction in those molecules is observed in the aboveground biomass of the plant due to a higher presence of senescing leaves and the redistribution of metabolites to reproductive organs [56]. The higher concentration of diterpene glycosides in stevia during early flowering is thought to be mainly developmentally regulated at the gene-expression level [57]. Similar regulation at the gene-expression level for the early-flowering accumulation of important metabolites is reported regarding the biosynthesis of menthol in *Mentha × piperita* L. [58] and methyleugenol in *Ocimum tenuiflorum* L. [59], among other Lamiaceae. For *Nepeta rtanjensis*, Diklić and Milojević pointed out the transcriptional regulation of the nepetalactone biosynthetic pathway related to the developmental stages of leaves during flowering [60]. Concerning *N. cataria*, Schultz et al. [28] hypothesize that a decrease in *E*,*Z*-nepetalactone levels after the appearance of floral buds may be a strategy to reduce repellency to pollinators, which may constitute a functional explanation for the ontogenetic regulation of iridoid terpene production. Future studies are needed to confirm those relationships as well as elucidate the developmental regulation of iridoid terpenes in *N. cataria.*

Along with the aforementioned environmental and ontogenetic influences, the productive performance of *N. cataria* plants was also influenced by propagule types in the present study. The seed-propagated plants used in this study originated from a cultivar producing stable contents of nepetalactone over multiple years [15,61], which, additionally, underwent multiple rounds of self-pollination to minimize the effects of genetic variation when compared to rooted stem cuttings. Although it is still possible that some of the results observed in this experiment are related to a higher variability in seeds, based on breeding background and plant uniformity, we believe that the variations observed in this experiment are better explained by physiological and morphological differences between the types of propagules.

In Pittstown, where environmental conditions seemed more appropriate for the productivity of nepetalactones, seed-propagated plants performed better than rooted cuttings in all harvests after the floral-bud stage for *E*,*Z*- and total nepetalactone contents, biomass, and total nepetalactone yield. In Upper Deerfield, the differences were less pronounced in terms of biomass and nepetalactone yield, but, in both locations, the peaks of *E*,*Z*- and total nepetalactone concentrations were higher in seed-propagated plants. In Pittstown, the peak nepetalactone yield in seed-propagated plants reached 1833.1 mg per plant, while cuttings reached a maximum yield of 1036.6 mg per plant. Overall, while plants propagated via seeds are known to form a well-defined tap root, the root system of stem cuttings is shallower and mainly composed of lateral roots [38,62]. Such characteristics can limit the ability of stem cuttings to absorb water from deeper portions of the soil, which can, in turn, influence gas exchange and hinder the assimilation of carbon.

*Camellia sinensis* (L.) Kuntze (tea plant) plants propagated via stem cuttings, for example, are reported to have lower xylem water potential and stomatal conductance than seedlings, especially during periods of lower precipitation, evidencing a lower tolerance to water stress [63]. Similarly, for *Eucalyptus* spp., 4-month-old seedlings had higher water potential and stomatal conductance than rooted stem cuttings of the same age, evidencing better tolerance to water stress, which led to a higher accumulation of biomass in the seminal propagules [64]. It is possible that lower assimilation of carbon not only hinders biomass accumulation but also affects the availability of precursors for terpene biosynthesis, which can explain the lower content of nepetalactone in stem cuttings in our study.

Another important distinction between stem cuttings and seedlings is the time to transition from vegetative to reproductive stages. Although some level of rejuvenation can be achieved in clonally propagated plants by specific methods, overall, sexually propagated plants have an ontogenetically defined juvenile phase [39], which delays the time of flowering in comparison to plants propagated by conventional rooted stem cuttings [65]. Because catnip plants produce the majority of nepetalactones during the flowering stage, reaching this stage too early, before the plants can accumulate enough biomass, can significantly reduce the productivity of nepetalactone per planted area. These differences were clearly observed in Pittstown when stem cuttings reached the flowering stage before seedlings and had lower productivity, but not in Upper Deerfield, where it seems that other environmental factors limited the productivity of plants propagated by both methods. Surprisingly, in contrast to nepetalactone, nepetalic acid, and nepetalactam, in both experimental sites, stem cuttings had higher contents of dihydronepetalactone than seedlings in almost all harvests. Although we were not able to precisely establish a likely cause for this trend, given the importance of this metabolite as an alternative insect repellent [11], future studies should further investigate the effects of agronomic practices on the accumulation of dihydronepetalactone.

Both rosmarinic and caffeic acid had similar patterns of accumulation through the growing season for both stem cuttings and seedlings and in both experimental locations, although higher concentrations were observed in Pittstown. The main trend was relatively stable concentrations throughout the growing season with peaks between 11 and 13 WAT. Although these phenolic acids have previously been identified in *N. cataria* extracts [14,66], and have been reported to be differentially accumulated across the crop’s successive harvests [15], little is known about the regulation of these molecules in the species. Duda et al. [23] have shown that rosmarinic acid is accumulated in higher amounts during the full-flowering stage rather than at the beginning of flowering in catnip, which is in agreement with our findings. Another possibility for the peak concentration of caffeic and rosmarinic acids at 13 WAT in Pittstown and 11 and 13 WAT in Upper Deerfield is the fact that these harvests took place at the end of September and the beginning of October (Table 2), after a month of low accumulated precipitation in September (Table 1). Contents of both rosmarinic and caffeic acid are reported to be higher during drought stress in plant species [67,68], and these molecules are thought to act as non-enzymatic antioxidants to mitigate the effects of reactive oxygen species originating from prolonged periods of stress [67,69].

Apigenin and luteolin had different patterns in Pittstown and Upper Deerfield, with clear differences between propagule types in Pittstown, where seedlings had relatively steady concentrations of both flavones across the growing season and stem cuttings had a much higher concentration at 3 WAT followed by a constant reduction in the flavone levels until the end of the season. Such differences may be related to stem cuttings undergoing more stress after transplanting than seedlings, which can increase the level of apigenin and luteolin since these compounds are known to be biotic and abiotic stress mediators in several plant species [70,71,72]. In Upper Deerfield, there were no differences between propagule types, but the plants tended to accumulate more flavones towards the end of the season, peaking at 13 and 15 WAT. In addition to their roles in plant stress physiology, luteolin and apigenin have been reported to accumulate in higher concentrations in plants with delayed senescence for environmental or genetic reasons [73,74], possibly due to their involvement in attenuating oxidative stress and maintaining membrane integrity for longer periods of time. In fact, we observed that in contrast to Pittstown, plants in Upper Deerfield took longer to flower, and the flowers did not senesce (Table 2) until they were killed by frost later in the season. We speculate that the accumulation of flavones at the end of the season is associated with the lack of appropriate environmental cues to trigger senescence in leaves and mainly inflorescences in Upper Deerfield. The specific mechanisms and the exact environmental cues associated with flower senescence in catnip remain largely unexplored and can contribute to the understanding of the environment–ontogeny interactions in the species, as well as provide strategic tools for growers to manipulate the phenology of the crop to achieve a higher productivity of metabolites of interest.

Interestingly, flavone glycosides had markedly different accumulation patterns than those of their aglycones and had more similar patterns to those of *E*,*Z*-nepetalactone and nepetalic acid for both propagule types, especially in Pittstown (Figure 5). Such patterns can point to the fact that the ontogenetic factors driving the accumulation of terpenes during flowering in catnip are similar to flavone glycosides. One possible explanation is that the beginning of flowering is when the plants reach peak photosynthetic activity before redirecting the carbon flow to reproductive organs; therefore. having a higher availability of carbon structures to act as building blocks of terpenes and the glycone portion of flavone glycosides. The reduction in flavone glycoside concentration after flowering may be related to the redistribution of such molecules to reproductive structures, such as that reported in *S. rebaudiana*’s diterpene glycosides [56]. Another possibility for flavone glycosides exhibiting a similar accumulation pattern to iridoid terpenes is that they share similar ecological functions in the plant, possibly as herbivore deterrents, since apigenin and luteolin glycosides have been reported to modify behaviors of insects and protect plants against herbivory [16,75]. Such convergent roles make it possible to infer that the ecological interactions that shaped catnip’s ontogenetic regulation of repellent terpenes also influenced the regulation of non-volatile defenses against herbivores.

**Table 1:** Weather variables in Pittstown and Upper Deerfield, State of New Jersey, United States, during the period of the experiment in 2019.

Site	Month	Ac Prec *	Avg RH	Avg Soil Temp 10 cm	Avg Temp	Max Temp	Min Temp	Avg SolarRad
		mm	%	°C				W/m^2^
Pittstown, NJ	June	132.8	70.7	20.3	20.5	25.7	15.5	254.2
	July	175.5	76.0	24.4	24.1	29.6	19.3	267.6
	Aug	99.3	77.4	23.3	22.1	27.6	17.7	230.0
	Sep	26.2	74.5	19.6	19.2	25.1	14.1	185.3
	Oct	137.4	77.3	14.9	13.6	18.4	9.1	110.4
	Nov	52.6	66.6	7.8	4.2	8.9	−0.5	95.7
Upper Deerfield, NJ	June	118.4	72.6	23.0	22.9	28.6	17.3	266.0
	July	119.9	78.0	26.8	26.0	31.9	20.6	269.0
	Aug	54.9	79.5	25.4	24.0	30.2	19.0	217.9
	Sep	10.2	74.2	22.5	21.3	28.6	14.7	193.0
	Oct	162.8	77.2	17.4	15.6	20.9	10.3	113.1
	Nov	32.0	69.4	9.5	5.7	11.7	0.1	98.2

* AcPrec: Accumulated Precipitation of the Month; AvgRH: Average Relative Humidity; AvgSoilTemp 10 cm: Average Soil Temperature at 10 cm depth; AvgTemp: Average Temperature; MaxTemp: Average Maximum Temperature; MinTemp: Average Minimum Temperature; AvgSolarRad: Average Solar Radiation. Source: Rutgers NJ Weather Network [76].

**Table 2:** Dates of harvest and developmental stages of *Nepeta cataria* L. plants propagated by seed or stem cutting in Pittstown and Upper Deerfield, New Jersey, United States, 2019.

Weeks after Transplanting		3	6	9	11	13	15
Date	Pittstown	15 July	6 August	28 August	11 September	25 September	9 October
	Upper Deerfield	30 July	19 August	9 September	23 September	7 October	21 October
Developmental-stage seed	Pittstown	Vegetative	Floral bud	Partial flowering	Full Flowering	Fruit set	Fruit Senescence
	Upper Deerfield	Vegetative	Vegetative	Floral bud	Partial flowering	Full flowering	Full flowering
Developmental-stage cutting	Pittstown	Vegetative	Partial flowering	Full flowering	Fruit set	Fruit Senescence	Fruit Senescence
	Upper Deerfield	Vegetative	Vegetative	Floral bud	Partial flowering	Full flowering	Fruit set

Agronomically, catnip plants propagated via seeds performed better than rooted stem cuttings, evidenced mainly by the superior productivity in Pittstown. In Upper Deerfield, although the peak productivities of nepetalactone were similar in stem cuttings and seedlings, stem cuttings only reached their peak productivity at 15 WAT, while seedlings reached their maximum production at 11 WAT. In Pittstown, similarly, peak stem-cutting productivity occurred at 13 WAT, while seedlings reached their peak at 9 WAT (Figure 2). This is very significant for catnip as it behaves as a semi-perennial crop; it regrows after a harvest and allows for two harvests within a season [15]. Because seed-propagated plants reach their peak productivity 4 weeks before stem cuttings and can, therefore, be harvested earlier, this allows more time for the crop to regenerate and accumulate more biomass for a second harvest. Although we did not assess a second harvest’s productivity in this study, this factor is of utmost importance for *N. cataria* cultivation, and the propagation via stem cuttings can significantly reduce its productivity in the course of one year.

This study provides important information on the agronomical aspects of catnips and ideal harvest times to maximize the productivity of nepetalactones in plants propagated via stem cuttings or seeds. In addition to the applied knowledge, an understanding of the ecophysiological patterns behind the interactions between cultivation conditions and plants’ secondary metabolism can help to unravel regulatory pathways that can be exploited to promote further increases in productivity and elucidate the ecological roles of plant metabolites.

## 4. Materials and Methods

### 4.1. Genotype and Plant Propagation

Catnip cultivar CR3 was the genotype used in this study. CR3 has been recently patented and is adapted for cultivation in North America, with an essential oil majorly composed of *E*,*Z*-nepetalactone, moderate amounts of *Z*,*E*-nepetalactone, and higher productivity than other commercially available lines [61].

CR3 plants were grown from self-pollinated seeds and maintained under greenhouse conditions for 6 months before being used as a source of propagules for the experiment. The plants were grown in peat-based growing medium with perlite and vermiculite within 2.8 L high-density polyethylene pots and watered once every two days until medium saturation. One of the plants was then selected and had the branches with open, mature flowers removed. Inflorescences containing immature, unopened flowers were covered with glassine bags (5 × 14 cm) to produce self-pollinated seeds. Once mature, the seeds were sown in semi-rigid, polypropylene, 128-cell plug trays (28 × 54 cm) filled with the previously described growing medium. The seedlings from this process were transplanted in 2.8 L high-density polyethylene pots and one plant was selected to repeat the process. The process was repeated four additional times. These multiple rounds of self-pollination were carried out to minimize the impact of genetic variation on the responses of seed-propagated plants. After the fifth consecutive generation of self-pollination, the seeds that originated from this process were grown, and one adult plant was selected to be cloned by single-node stem cuttings. A total of 25 stem cuttings were taken from the plant, of which 22 rooted and were transplanted into 2.8 L pots. Those pots were then placed in a separate room in the greenhouse, isolated from other genotypes, and the clones were allowed to cross-pollinate amongst themselves. The seeds collected from these cross-pollinated clones were used to produce the seedlings assessed in the experiment. The clones were then clipped at about 10 cm from the soil level and allowed to regrow for 30 days. After 30 days, before the plants flowered, stem cuttings were taken from the clones, and the ones that were rooted were used in the experiment.

The seeds collected from the clones were sown in 128-cell plug trays (cell volume approximately 22 cm^3^, 5 cm tall) filled with peat-based growing medium containing perlite and vermiculite. The seeds were gently covered with the growing medium and kept under intermittent misting (5 s every 30 min) until germination. After germination, the trays containing the seedlings were kept under greenhouse conditions and watered manually, once a day, until medium saturation. One week after germination, extra seedlings were removed to allow for the growth of a single seedling per cell. After 45 days from sowing, seedlings with five to six pairs of leaves and with a height of 15 to 20 cm (soil level to shoot apex) were used in the field experiment.

Single-node stem cuttings were taken from the middle section of vegetative branches from cultivar CR3 clones (clones from a parent plant that underwent six consecutive generations of self-pollination). Stem cuttings were 7 cm long, with a beveled cut at the base and a straight cut at the apex. Two leaves, each reduced to approximately half of its original area, were left at the apical portion of the cuttings. Cutting bases were kept in distilled water until planting to avoid dehydration. The propagules were planted, one per cell, in the same cell plug trays described for the seedlings. The bases of the cuttings were inserted, at a depth of 3 cm, into the growing medium, and the trays were kept under intermittent misting (5 s every 30 min) for 30 days and then kept under greenhouse conditions with manual watering every other day until soil saturation. Sixty days after planting, the rooted cuttings with new sprouts of 10 to 15 cm height were selected for the field experiment. The plants were left under field conditions and were manually irrigated daily for 7 days for acclimatization before transplanting.

### 4.2. Field Conditions, Transplanting and Experimental Design

**Figure 6:**
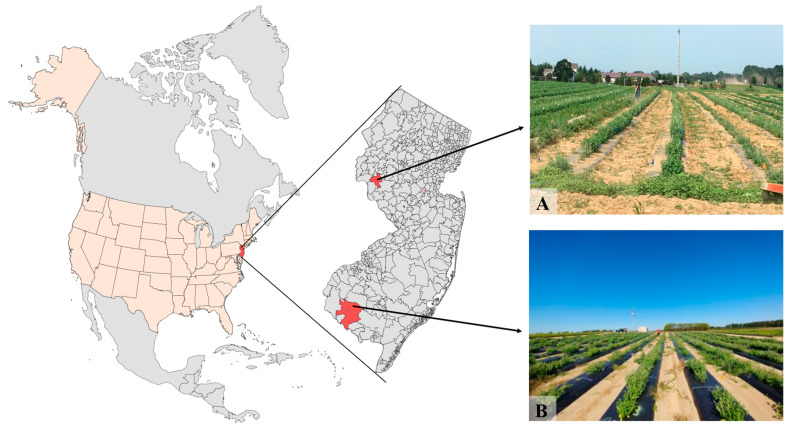
Geographical location of the state of New Jersey in North America and the two experimental sites in the cities of Pittstown, NJ (**A**), and Upper Deerfield, NJ (**B**).

The experiments were conducted in 2019 in two locations in the state of New Jersey, United States: Pittstown and Upper Deerfield. The geographical locations of the two experimental sites are depicted in Figure 6. The Pittstown site is located in the northwestern portion of the state of New Jersey at 40°33′26.7″ N; 74°57′37.7″ W and 116 m altitude, with soil characterized as silt loam. Upper Deerfield is located in the southern region of New Jersey at 39°31′10.8″ N; 75°12′13.5″ W and 28 m altitude, with Aura sandy loam soil. The weather variables in each site during the period of the experiment are presented in Table 1.

**Figure 7:**
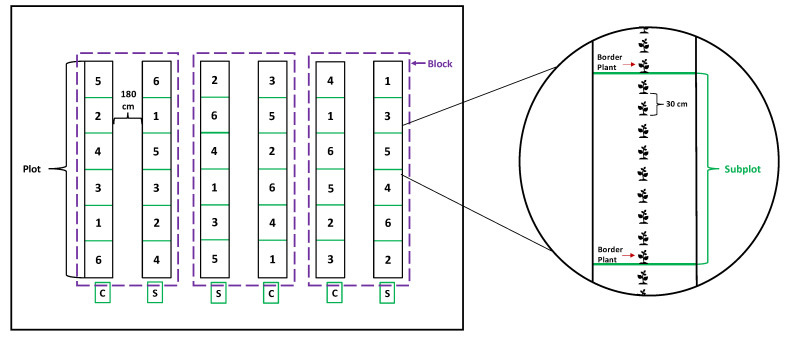
Schematic diagram of the split-plot design within a completely randomized block design to assess the effects of types of propagules (whole-plot) and harvesting times (split-plot) on the productive and phytochemical profiles of catnip (*Nepeta cataria* L.). C: rooted stem cuttings; S: seedlings. The numbers in the subplots (split-plots) represent the order of the harvests: 1, 2, 3, 4, 5, and 6 represent 3, 6, 9, 11, 13, and 15 weeks after transplanting, respectively.

The experiment was arranged in a completely randomized block design, with three blocks. The whole-plot treatments were the types of propagules (rooted cuttings or seeds), and the split-plot treatments were six harvest dates at 3, 6, 9, 11, 13, and 15 weeks after transplanting. Each subplot was 2.7 m in length and contained eight plants to be harvested, and there was a border (buffer) plant at each end of the plot. Each of the subplots was harvested at different times within the season, which were randomized within the plots (Figure 7). The same experimental design was used at the Pittstown and Upper Deerfield sites.

### 4.3. Determination of Developmental Stages, Harvesting and Postharvest Handling

At each harvest time, the developmental stage of the plants was assessed by observing the presence or stage of flowers, fruits, and seeds in the plants and estimating the percentage of plants in each plot with similar characteristics. The vegetative stage was represented by the absence of flowers, fruits, and seeds. The floral-bud stage was determined as the stage when more than 50% of the plants showed floral structures, but with no open flowers (no apparent petals). Partial flowering was defined as the stage when less than 50% of the plants had the majority of their flowers open. Full flowering was defined as the state when more than 50% of the plants had the majority of their flowers open, while the fruit set was defined as the stage when the nutlets were visible (protuberant) and green color in more than 50% of the plants. Fruit senescence was defined when more than 50% of the plants showed the exterior layer of the nutlets to be dried and brown in color. The dates of harvest and respective developmental stages of plants propagated by seeds and stem cuttings in both experimental sites are presented in Table 2.

The plants were harvested by clipping the stems about 10 cm above soil level with hedge shears, and the aboveground biomass was collected in brown paper bags (113.6 L volume). The aboveground biomass was dried to constant weight at a temperature of 37 °C in a tobacco dryer with forced air circulation (MarCo Manufacturing Company LLC, Bennettsville, SC, USA). After drying, the main stems of the plants were discarded, and the aboveground dry biomass was assessed. The dried plants were then ground to a fine powder with a DCG-12BC coffee grinder (Cuisinart^®^, East Windsor, NJ, USA) for phytochemical analyses.

### 4.4. Phytochemical and Statistical Analyses

Catnip terpenes were analyzed using ultra-high-performance liquid chromatography coupled with triple quadrupole mass spectrometry (UHPLC-QqQ-MS), as described by Patel et al. [10], with slight modifications. Briefly, approximately 100 mg of ground aboveground biomass was diluted in 10 mL of reagent alcohol (90% ethanol, 5% methanol, 5% isopropyl alcohol) and the mixture was sonicated for 5 min in an ultrasonic bath (M5800 Ultrasonic Cleaner, Branson Ultrasonics, Brookfield, CT, USA). After remaining at room temperature for 12 h, 40 μL of the supernatant was added to 960 μL of ACS-grade methanol and vortexed for homogenization. This extract was centrifuged at t 10,000× *g* for 10 min (AccuSpin Micro17 centrifuge, Fisher Sci, Hampton, NH, USA), and 500 μL of the supernatant was added to HPLC vials and stored at −20 °C until phytochemical analysis.

A 1290 Infinity II UHPLC (Agilent Technologies, Santa Clara, CA, USA) and a Waters Acquity UPLC BEH C18, 2.1 × 50 mm, 1.7 μm (Milford, MA, USA) column were used for the separation of the compounds. Mobile phase A was LC-MS-grade water with 0.1% of LC-MS-grade formic acid, and Mobile phase B was LC-MS-grade acetonitrile with 0.1% of LC-MS-grade formic acid. The elution gradient was the following: 0–30% B for 0–0.2 min, 30–45% B for 0.2–4 min, isocratic 45% B for 4–4.5 min, 45–100% B for 4.5–4.6 min, and 100% B for 4.6–6 min. The column was washed with 100% B for 1 min and then the gradient was switched back to 10% B for 1 min. The column temperature was kept at 30 °C and the autosampler temperature was maintained at 4 °C throughout the sample run. The eluent before 1 min and after 4.5 min was eluted to waste. The injection volume was 0.7 µL and the flow rate was 0.4 mL/min, for a total runtime of 7 min. Each injection was followed by a wash with 70% methanol for 3 s.

For mass spectra analysis, a 6470 triple quadrupole mass spectrometer featuring an electrospray ionization (ESI) source (Agilent Technologies, Santa Clara, CA, USA) was used. High-purity nitrogen, supplied by a NitroFlow 60NA nitrogen generator (Parker Balston, Cleveland, OH, USA), was used as the nebulizing, sheath, and drying gas. The nebulizer pressure was at 50 psi, the sheath gas was set at 250 °C with a flow rate of 12 L/min, and the drying gas was kept at 300 °C with a flow rate of 13 L/min. The capillary and nozzle voltages were set at 3000 V and 1500 V, respectively, while the multiplier voltage was zero. Positive multiple reaction monitoring (MRM) mode was used as scanning mode, with parameters for nepetalic acid, dihydronepetalactone, and nepetalactone being the same as those described by Patel et al. [10]. For nepetalactones, MRM parameters are presented in Table 3.

MassHunter Workstation Data Acquisition (version B.10.0), Qualitative Analysis (version B.10.0), and Quantitative Analysis (version B.10.0) (Agilent Technologies, Santa Clara, CA, USA) were used for data acquisition and processing. The concentration of each compound was calculated with a calibration curve, with reference standards of nepetalactones, nepetalic acid, nepetalactam, and dihydronepetalactone, and is expressed as mg/100 g dry weight.

Polyphenols in catnip tissues were analyzed via UHPLC with diode-array detection (DAD) (Agilent 1290 Infinity II) according to the method described by Gomes et al. [15]. The column and mobile phases for polyphenol analysis were the same as those previously described for terpene separation. The elution gradient was the following: 3–20% B for 0–6 min, 20–45% B for 6–11 min, 45–100% B for 11–11.1 min, and isocratic 100% B for 11.1–11.5 min. After this period, the column was washed for 1 min with Mobile phase B and then equilibrated with 3% B. The autosampler temperature was the same as that described for the terpene analysis. The eluent before 2.8 min and after 11 min was eluted to waste. The flow rate of the method was 0.5 mL per minute, for a total runtime of 11.50 min, with 1.5 min of post-time for column equilibration. The injection volume was 2 μL and, after injection, the needle was washed as previously described for terpene analysis. The DAD was set to the wavelengths of 254 nm, 320 nm, 370 nm, and 500 nm with a spectrum scan range of 190 nm to 400 nm.

For the initial identification of the polyphenols, a 6546 quadrupole time-of-flight mass spectrometer (QTOF-MS) coupled to a dual jet stream (AJS) electrospray ionization (ESI) source (Agilent Technologies, Santa Clara, CA, USA) was employed. High-purity nitrogen was used as the drying, sheath, and nebulizing gas. The drying and sheath were set to a flow rate of 13 L/minute and 10 L/minute, respectively, and both were kept at 250 °C. The nebulizer pressure was 30 psi. The capillary and nozzle voltages were both 5.0 kV, and the samples were analyzed in positive ion mode. The identification of phenolic acids and flavones was confirmed by authenticated standards and flavone glycosides were tentatively identified via high-resolution mass spectrometry (HRMS).

Polyphenols were quantified at 320 nm. Calibration curves for caffeic acid, rosmarinic acid, luteolin, and apigenin were established using commercial standards. Luteolin glycosides and apigenin glycosides quantification was based on correction factors of molecular weight ratio to their respective aglycones, as described by Reichert et al. [14]. Data acquisition and analysis were performed via the same software previously described for terpene analysis. A representative chromatogram of the polyphenolic composition of *N. cataria* methanolic extract as well as the mass spectra of the identified compounds are presented in Supplementary Figures S1–S3.

The homogeneity of variances and normal distribution of residuals was assessed by the Brown–Forsythe and Kolmogorov–Smirnov tests, respectively, and data of variables that did not meet the assumptions of analysis of variance (ANOVA) were transformed by *Y* = *Log*2(*Y*) or *Y* = *sin*(*Y*) (Supplementary Tables S13 and S14). Split-plot ANOVA was performed on the data, with the whole-plot factor being the type of propagule and the split-plot factor being the time of harvest. When differences were statistically significant (*p* ≤ 0.05), Tukey’s post hoc test was applied. GraphPad Prism version 9.0.0 for Windows was used for the assessment of ANOVA assumptions, data transformation, and graphical representation. Statistical software ASSISTAT (Version 7.7) [77] was used to perform the Split-plot ANOVA and post hoc test.

## 5. Conclusions

Propagule types affect the phytochemistry and productivity of *Nepeta cataria* plants as well as their response to seasonality factors in different experimental locations. When field conditions were conducive to a higher accumulation of biomass and nepetalactones (Pittstown experimental site), the ideal time to harvest catnip propagated via seeds for the maximized productivity of nepetalactones (mg per plant) was between 9 and 11 weeks after transplanting, while rooted stem cuttings reached maximum productivity at 13 weeks after transplanting. When field conditions were not ideal for the high productivity of catnip (Upper Deerfield), the peak nepetalactone productivity of seed-propagated plants took place 11 weeks after transplanting, and stem-cutting-propagated plants reached their maximum productive capacity 15 weeks after transplanting. The main factors limiting catnip productivity associated with location are high temperatures and low precipitation. Maximum contents (mg/100 g of plant material) of nepetalactone occur during the floral-bud to partial-flowering stages and, in Pittstown, where plants produced more, stem cuttings reached peak contents earlier than seedlings. Rosmarinic and caffeic acid accumulation is associated with prolonged periods of low accumulated precipitation. Flavone glycosides have similar accumulation patterns to nepetalactones across the growing season in two distinct growing locations, suggesting similar ecological roles and regulation mechanisms. Overall, seed-propagated plants have superior agronomic performance than stem cuttings due to both their higher biomass accumulation in conducive environments and reaching the peak productivity of nepetalactones, on average, four weeks earlier.

## Figures and Tables

**Table 3:** Multiple reaction monitoring (MRM) parameters for *Z*,*E-* and *E*,*Z*-nepetalactone (NL) isomers.

Compounds	RT * (min)	Precursor (*m*/*z*)	Frag	Quant (*m*/*z*)	CE (eV)	Qual 1 (*m*/*z*)	CE (eV)	Ratio	Qual 2 (*m*/*z*)	CE (eV)	Ratio
*Z*,*E*-NL	3.57	167.0	95	55.1	34	77.0	38	0.99	43.1	30	0.87
*E*,*Z*-NL	3.89	167.0	70	77.0	41	43.1	30	0.78	81.1	13	0.69

* RT: retention time; Frag: fragmentor voltage; Quant: quantifier ion; Qual: qualifier ion; CE: collision energy. Qualifier ion ratio is the peak area of the qualifier ion divided by the peak area of the associated quantifier ion.

## Data Availability

Data associated with this study can be found in Supplementary Materials. Additional data are available upon request from the corresponding author.

## Supplementary Materials

The following supporting information can be downloaded at: https://www.mdpi.com/article/10.3390/molecules29092001/s1, Table S1: Analysis of variance (ANOVA) table for contents of *E*,*Z*-nepetalactone, *Z*,*E*-nepetalactone, total nepetalactones, nepetalic acid, dihydronepetalactone, nepetalactam, biomass accumulation (biomass) and total nepetalactone yield in catnip plants propagated by different methods and harvested at different times within the growing season. Pittstown, State of New Jersey, United States; Table S2: Analysis of variance (ANOVA) table for contents of *E*,*Z*-nepetalactone, *Z*,*E*-nepetalactone, total nepetalactones, nepetalic acid, dihydronepetalactone, nepetalactam, biomass accumulation (biomass) and total nepetalactone yield in catnip plants propagated by different methods and harvested at different times within the growing season. Upper Deerfield, State of New Jersey, United States; Table S3: Unfolding of interaction effects between propagation methods and harvest times on contents of *E*,*Z*-nepetalactone, *Z*,*E*-nepetalactone, total nepetalactones, nepetalic acid, dihydronepetalactone, nepetalactam, biomass accumulation and total nepetalactone yield in catnip plants. Pittstown, State of New Jersey, United States; Table S4: Unfolding of interaction effects between propagation methods and harvest times on contents of *E*,*Z*-nepetalactone, *Z*,*E*-nepetalactone, total nepetalactones, nepetalic acid, dihydronepetalactone, nepetalactam, biomass accumulation and total nepetalactone yield in catnip plants. Upper Deerfield, State of New Jersey, United States; Table S5: Analysis of variance (ANOVA) table for contents of caffeic acid, rosmarinic acid, luteolin and apigenin in catnip plants propagated by different methods and harvested at different times within the growing season. Pittstown, State of New Jersey, United States; Table S6: Analysis of variance (ANOVA) table for contents of caffeic acid, rosmarinic acid, luteolin, and apigenin in catnip plants propagated by different methods and harvested at different times within the growing season. Upper Deerfield, State of New Jersey, United States; Table S7: Unfolding of interaction effects between propagation methods and harvest times on contents of caffeic acid, rosmarinic acid, luteolin, and apigenin in catnip plants. Pittstown, State of New Jersey, United States; Table S8: Unfolding of interaction effects between propagation methods and harvest times on contents of caffeic acid, rosmarinic acid, luteolin, and apigenin in catnip plants. Upper Deerfield, State of New Jersey, United States; Table S9: Analysis of variance (ANOVA) table for contents of luteolin glucoside, apigenin glucoside, luteolin glucuronide, apigenin glucuronide, luteolin diglucuronide, and apigenin diglucuronide in catnip plants propagated by different methods and harvested at different times within the growing season. Pittstown, State of New Jersey, United States; Table S10: Analysis of variance (ANOVA) table for contents of luteolin glucoside, apigenin glucoside, luteolin glucuronide, apigenin glucuronide, luteolin diglucuronide, and apigenin diglucuronide in catnip plants propagated by different methods and harvested at different times within the growing season. Upper Deerfield, State of New Jersey, United States; Table S11: Analysis of variance (ANOVA) table for contents of luteolin glucoside, apigenin glucoside, luteolin glucuronide, apigenin glucuronide, luteolin diglucuronide, and apigenin diglucuronide in catnip plants propagated by different methods and harvested at different times within the growing season. Pittstown, State of New Jersey, United States; Table S12: Analysis of variance (ANOVA) table for contents of luteolin glucoside, apigenin glucoside, luteolin glucuronide, apigenin glucuronide, luteolin diglucuronide, and apigenin diglucuronide in catnip plants propagated by different methods and harvested at different times within the growing season. Upper Deerfield, State of New Jersey, United States; Figure S1: Representative UHPLC-DAD chromatogram (320 nm) of phenolic compounds in the methanolic extracts of *Nepeta cataria* L. aerial parts; Figure S2: Mass spectra of caffeic acid (A), rosmarinic acid (B), luteolin (C), and apigenin (D) in methanolic extracts of *Nepeta cataria* L. aerial parts; Figure S3: Mass spectra of apigenin glucoside (A), luteolin glucoside (B), apigenin glucuronide (C), luteolin glucuronide (D), apigenin diglucuronide (E), and luteolin diglucuronide (F) in methanolic extracts of *Nepeta cataria* L. aerial parts; Table S13: Results of Brown–Forsythe (homogeneity of variances) and Kolmogorov–Smirnov (normal distribution of residuals) tests on original and transformed data of different variables of catnip plants propagated by different methods and harvested at different times within the growing season. Pittstown, State of New Jersey, United States; Table S14: Results of Brown–Forsythe (homogeneity of variances) and Kolmogorov–Smirnov (normal distribution of residuals) tests on original and transformed data of different variables of catnip plants propagated by different methods and harvested at different times within the growing season. Upper Deerfield, State of New Jersey, United States.
