# Supplementary material for: Implications of the Propagation Method for the Phytochemistry of Nepeta cataria L. throughout a Growing Season
Source: Molecules. 2024 Apr 26;29(9):2001. doi: 10.3390/molecules29092001 (PMC11085440; doi:10.3390/molecules29092001)
Supplement: Supplementary file 1 [file molecules-29-02001-s001.zip › molecules-2958942-supplementary.pdf]

molecules

MDPI

Supplementary materials

Implications of the Propagation Method for thePhytochemistry of Nepeta cataria L. throughout a Growing Season

Erik Nunes Gomes [1,2*], Bo Yuan[1], Harna K. Patel [1], Anthony Lockhart [1,3,4], Christian A. Wyenandt [1,5], Qingli Wu [1,3,4], James E. Simon [1,3,4*]

[1] New Use Agriculture and Natural Plant Products Program, Department of Plant Biology, Rutgers University, New Brunswick, NJ 08901, USA
[2] Federal Agency for Support and Evaluation of Graduate Education (CAPES), Ministry of Education of Brazil, Brasilia 70040-020, DF, Brazil
[3] Rutgers Core Facility for Natural Products and Bioanalysis, Rutgers University, New Brunswick, NJ 08901, USA
[4] Department of Medicinal Chemistry, Ernest Mario School of Pharmacy, Rutgers University, Piscataway, NJ 08854, USA
[5] New Jersey Agricultural Experiment Station, Rutgers Agricultural Research and Extension Center (RAREC), Department of Plant Biology, Rutgers University, Bridgeton, NJ 08302, USA

* Correspondence: erik.gomes@rutgers.edu (E.N.G.); jimsimon@sebs.rutgers.edu (J.E.S.)

Table S1. Analysis of variance (ANOVA) table for contents of *E*,*Z*-nepetalactone (*E*,*Z*-NL), *Z*,*E*-nepetalactone (*Z*,*E*-NL), total nepetalactones (Total NL), nepetalic acid (NA), dihydronepetalactone (DHNL), nepetalactam (NT), biomass accumulation (biomass) and total nepetalactone yield (NL yield) in catnip plants propagated by different methods and harvested at different times within the growing season. Pittstown, State of New Jersey, United States.

Source of variation	DF	Sum of squares							
		E,*Z*-NL	*Z*,*E*-NL	Total NL	NA	DHNL	NT	Biomass	NL yield
Blocks	2	17357.2^{ns}	52.1^{ns}	19371.8^{ns}	6536.9**	439.5^{ns}	0.65*	350.5^{ns}	168699.4^{ns}
Propagule (p)	1	566399.8**	5.3^{ns}	601921.1**	21098.1**	30040.9**	0.02^{ns}	16628.1*	3714516.1*
Whole-Plot error	2	2595.1	3.0	2954.5	42.2	167.3	0.02	1555.2	133410.8
Weeks (w)	5	3053547.1**	21565.4**	3656447.8**	695683.1**	74016.1**	727.3**	132262.9**	5846481.9**
Interaction p×w	5	427632.2**	2988.3**	496172.9**	27671.0**	78367.2**	3.2**	10318.4**	3092197.7**
Split-Plot Error	20	24505.1	315.3	31715.5	6724.2	1044.5	1.7	9293.8	700279.2
Total	35	4092036.5	24929.4	4808583.7	757755.7	184075.5	732.9	170408.9	13655585.3

* Statistically significant ($p \leq 0.05$); ** Statistically significant ($p \leq 0.01$); ^{ns} Not statistically different ($p > 0.05$); DF: degrees of freedom.

molecules

MDPI

Table S2. Analysis of variance (ANOVA) table for contents of *E*,*Z*-nepetalactone (*E*,*Z*-NL), *Z*,*E*-nepetalactone (*Z*,*E*-NL), total nepetalactones (Total NL), nepetalic acid (NA), dihydronepetalactone (DHNL), nepetalactam (NT), biomass accumulation (biomass) and total nepetalactone yield (NL yield) in catnip plants propagated by different methods and harvested at different times within the growing season. Upper Deerfield, State of New Jersey, United States.

Source of variation	DF	Sum of squares							
		E,*Z*-NL	*Z*,*E*-NL	Total NL	NA	DHNL	NT	Biomass	NL yield
Blocks	2	42779.1*	56.4^{ns}	50494.4*	7252.8^{ns}	235.4^{ns}	0.21^{ns}	37.1^{ns}	12786.0^{ns}
Propagule (p)	1	62451.5*	18.4^{ns}	66455.4*	129535.9**	5538.1*	0.13^{ns}	280.6^{ns}	426.6^{ns}
Whole-Plot error	2	1905.1	20.2	2474.8	814.1	284.3	0.14	548.4	34962.7
Weeks (w)	5	1948263.8**	5106.9**	2143273.9**	162753.4**	19759.9**	99.5**	27072.9**	1455585.9**
Interaction p×w	5	905149.9**	836.5**	917602.4**	107678.8**	4311.8**	1.68**	141.0^{ns}	237512.6**
Split-Plot Error	20	58673.5	215.8	68095.2	7525.7	341.6	0.57	2093.6	140383.8
Total	35	3019222.9	6254.4	3248396.4	415560.6	30471.1	102.3	30173.5	1881657.7

* Statistically significant ($p \leq 0.05$); ** Statistically significant ($p \leq 0.01$); ^{ns} Not statistically different ($p > 0.05$); DF: degrees of freedom.

molecules MDPI

Table S3. Unfolding of interaction effects between propagation methods and harvest times on contents of *E*,*Z*-nepetalactone, *Z*,*E*-nepetalactone, total nepetalactones, nepetalic acid, dihydronepetalactone, nepetalactam, biomass accumulation and total nepetalactone yield (NL yield) in catnip plants. Pittstown, State of New Jersey, United States.

**E,Z-nepetalactone (mg per 100 g)[1]**						
Propagule	*Weeks after planting*					
	3	6	9	11	13	15
Seed	304.8 aD	1245.7 aA	1160.6 aA	802.4 aB	660.5 aC	640.6 aC
Cutting	335.6 aC	1126.9 bA	645.4 bB	344.6 bC	630.6 aB	226.2 bD
**Z,E-nepetalactone (mg per 100 g)**						
Propagule	*Weeks after planting*					
	3	6	9	11	13	15
Seed	4.9 aC	38.6 bB	75.1 aA	34.5 aB	10.6 aC	14.9 aC
Cutting	3.3 aD	75.7 aA	61.1 bB	14.7 bC	9.5 aCD	9.6 aCD
**Total nepetalactones (mg per 100 g)**						
Propagule	*Weeks after planting*					
	3	6	9	11	13	15
Seed	314.3 aD	1305.4 aA	1268.3 aA	861.3 aB	679.9 aC	668.6 aC
Cutting	341.1 aCD	1223.3 bA	721.2 bB	367.1 bC	649.3 aB	244.2 bD
**Nepetalic acid (mg per 100 g)**						
Propagule	*Weeks after planting*					
	3	6	9	11	13	15
Seed	279.9 aD	614.3 aA	451.2 aB	397.5 aC	209.8 aE	190.7 aE
Cutting	304.6 aC	520.5 bA	438.9 aB	261.3 bC	202.8 aD	124.7 bE
**Dihydronepetalactone (mg per 100 g)**						
Propagule	*Weeks after planting*					
	3	6	9	11	13	15
Seed	184.4 aA	81.8 bD	149.7 bB	147.9 bB	101.1 aC	86.5 bCD
Cutting	90.1 bD	204.4 aB	343.8 aA	175.8 aC	98.1 aD	185.9 aBC
**Nepetalactam (mg per 100 g)**						
Propagule	*Weeks after planting*					
	3	6	9	11	13	15
Seed	6.1 aB	13.5 bA	5.9 aB	1.8 aC	0.8 aD	0.7 bD
Cutting	4.9 bB	14.1 aA	5.7 aB	2.2 aC	0.7 aD	1.4 aD
**Biomass (g per plant)**						
Propagule	*Weeks after planting*					
	3	6	9	11	13	15
Seed	24.4 aC	51.4 aC	143.7 aB	211.1 aA	194.5 aAB	197.8 aAB
Cutting	7.9 aD	62.5 aCD	82.8 bBC	119.8 bAB	159.9 aA	131.9 bAB
**Total nepetalactone yield (mg per plant)**						
Propagule	*Weeks after planting*					
	3	6	9	11	13	15
Seed	77.1 aD	668.3 aC	1833.1 aA	1821.0 aA	1323.3 aB	1319.9 aB
Cutting	26.9 aC	770.4 aAB	595.4 bAB	438.26 bBC	1036.5 aA	320.4 bBC

[1]Means followed by the same letter do not differ statistically according to the Tukey test at the 5% probability level. Lowercase letters indicate differences between methods of propagation within the same harvest date (column) and uppercase letters indicate differences among different dates of harvesting within the same propagation method (row).

molecules

MDPI

Table S4. Unfolding of interaction effects between propagation methods and harvest times on contents of *E*,*Z*-nepetalactone, *Z*,*E*-nepetalactone, total nepetalactones, nepetalic acid, dihydronepetalactone, nepetalactam, biomass accumulation and total nepetalactone yield (NL yield) in catnip plants. Upper Deerfield, State of New Jersey, United States.

**_E,Z_-nepetalactone (mg per 100 g)[1]**						
Propagule	*Weeks after planting*					
	3	6	9	11	13	15
Seed	305.7 bC	399.4 bBC	1157.9 aA	1192.0 aA	501.9 bB	445.7 bB
Cutting	663.8 aB	491.5 aC	735.1 bB	950.7 bA	949.5 aA	711.8 aB
**_Z,E_-nepetalactone (mg per 100 g)**						
Propagule	*Weeks after planting*					
	3	6	9	11	13	15
Seed	7.8 bD	11.8 aD	28.6 bC	36.1 aBC	38.4 aAB	45.9 aA
Cutting	23.2 aD	9.7 aE	41.0 aAB	33.3 aBC	25.6 bCD	44.3 aA
**Total nepetalactones (mg per 100 g)**						
Propagule	*Weeks after planting*					
	3	6	9	11	13	15
Seed	318.5 bC	416.6 aBC	1213.9 aA	1247.7 aA	548.3 bB	510.4 bB
Cutting	698.0 aB	508.3 aC	798.6 bB	997.1 bA	993.1 aA	775.9 aB
**Nepetalic acid (mg per 100 g)**						
Propagule	*Weeks after planting*					
	3	6	9	11	13	15
Seed	383.2 bB	280.5 bC	264.6 bCD	475.9 aA	215.2 bD	363.3 aB
Cutting	629.8 aA	379.2 aD	407.3 aCD	437.4 bBC	471.2 aB	377.6 aD
**Dihydronepetalactone (mg per 100 g)**						
Propagule	*Weeks after planting*					
	3	6	9	11	13	15
Seed	75.3 aA	72.3 bA	70.7 bA	48.6 bB	36.0 bC	41.7 aBC
Cutting	86.6 aC	132.6 aA	112.6 aB	66.1 aD	61.9 aD	33.5 aE
**Nepetalactam (mg per 100 g)**						
Propagule	*Weeks after planting*					
	3	6	9	11	13	15
Seed	2.3 aC	4.9 aB	5.5 aA	2.6 bC	1.6 aD	0.8 aE
Cutting	1.6 bD	4.7 aB	5.2 aA	3.2 aC	1.3 aDE	1.1 aE
**Biomass (g per plant)#**						
Propagule	*Weeks after planting*					
	3	6	9	11	13	15
Seed	2.1	8.3	25.9	47.3	52.3	84.7
Cutting	1.8	4.4	18.0	35.0	50.0	77.8
**Total nepetalactone yield (mg per plant)**						
Propagule	*Weeks after planting*					
	3	6	9	11	13	15
Seed	6.6 aC	34.4 aC	310.8 aB	591.7 aA	288.1 bB	430.1 bAB
Cutting	12.4 aD	22.3 aD	143.5 bCD	348.4 bBC	494.9 aAB	598.9 aA

[1]Means followed by the same letter do not differ statistically according to the Tukey test at the 5% probability level. Lowercase letters indicate differences between methods of propagation within the same harvest date (column) and uppercase letters indicate differences among different dates of harvesting within the same propagation method (row).#Interaction effect not statistically significant.

molecules

MDPI

Table S5. Analysis of variance (ANOVA) table for contents of caffeic acid (Ca), rosmarinic acid (Ra), luteolin (Lu) and apigenin (Ap) in catnip plants propagated by different methods and harvested at different times within the growing season. Pittstown, State of New Jersey, United States.

Source of variation	DF	Sum of Squares			
		Ca	Ra	Lu	Ap
Blocks	2	2.5^{ns}	188.4^{ns}	8.9^{ns}	1.1^{ns}
Propagule (p)	1	437.9**	40997.8**	94.9*	21.6^{ns}
Whole-Plot error	2	1.2	442.7	6.1	2.8
Weeks (w)	5	201.2**	304389.7**	145.8**	88.1**
Interaction p×w	5	15.7^{ns}	104049.1**	371.5**	76.6**
Split-Plot Error	20	58.3	1969.9	83.8	17.9
Total	35	716.8	452037.6	711.1	208.1

* Statistically significant ($p \leq 0.05$); ** Statistically significant ($p \leq 0.01$); ^{ns} Not statistically different ($p > 0.05$); DF: degrees of freedom.

Table S6. Analysis of variance (ANOVA) table for contents of caffeic acid (Ca), rosmarinic acid (Ra), luteolin (Lu) and apigenin (Ap) in catnip plants propagated by different methods and harvested at different times within the growing season. Upper Deerfield, State of New Jersey, United States.

Source of variation	DF	Sum of Squares			
		Ca	Ra	Lu	Ap
Blocks	2	19.4^{ns}	610.9^{ns}	7.6^{ns}	6.2^{ns}
Propagule (p)	1	0.1^{ns}	6579.6^{ns}	8.0^{ns}	4.1^{ns}
Whole-Plot error	2	2.9	1501.1	1.3	0.9
Weeks (w)	5	122.7**	73286.8**	195.2**	70.6**
Interaction p×w	5	47.58^{ns}	7704.7**	42.4^{ns}	24.5^{ns}
Split-Plot Error	20	87.0	7299.1	78.9	38.7
Total	35	279.8	96982.3	333.5	145.1

* Statistically significant ($p \leq 0.05$); ** Statistically significant ($p \leq 0.01$); ^{ns} Not statistically different ($p > 0.05$); DF: degrees of freedom.

molecules

MDPI

Table S7. Unfolding of interaction effects between propagation methods and harvest times on contents of caffeic acid (Ca), rosmarinic acid (Ra), luteolin (Lu) and apigenin (Ap) in catnip plants. Pittstown, State of New Jersey, United States.

Caffeic acid (mg per 100 g)#						
Propagule	*Weeks after planting*					
	3	6	9	11	13	15
Seed	12.6	16.6	14.5	14.9	20.4	14.2
Cutting	6.8	7.8	5.9	7.9	13.6	9.1
**Rosmarinic acid (mg per 100 g) [1]**						
Propagule	*Weeks after planting*					
	3	6	9	11	13	15
Seed	2.7 aE	8.7 aE	36.6 aD	192.7 aB	410.4 aA	127.3 aC
Cutting	2.2 aD	24.4 aCD	35.2 aBC	59.6 bB	129.8 bA	122.2 aA
**Luteolin (mg per 100 g)**						
Propagule	*Weeks after planting*					
	3	6	9	11	13	15
Seed	7.1 bAB	2.8 bB	7.4 bAB	8.4 aA	7.5 aAB	8.2 aA
Cutting	16.8 aA	15.9 aA	12.1 aA	5.6 aB	5.8 aB	4.6 bB
**Apigenin (mg per 100 g)#**						
Propagule	*Weeks after planting*					
	3	6	9	11	13	15
Seed	3.1 bA	2.5 bA	2.6 aA	2.9 aA	3.2 aA	2.1 aA
Cutting	10.2 aA	5.8 aB	2.1 aC	3.9 aBC	1.6 aC	2.1 aC

[1]Means followed by the same letter do not differ statistically according to the Tukey test at the 5% probability level. Lowercase letters indicate differences between methods of propagation within the same harvest date (column) and uppercase letters indicate differences among different dates of harvesting within the same propagation method (row). #Interaction effect not statistically significant.

Table S8. Unfolding of interaction effects between propagation methods and harvest times on contents of caffeic acid (Ca), rosmarinic acid (Ra), luteolin (Lu) and apigenin (Ap) in catnip plants. Upper Deerfield, State of New Jersey, United States.

Caffeic acid (mg per 100 g)#						
Propagule	*Weeks after planting*					
	3	6	9	11	13	15
Seed	9.3	11.2	7.3	13.8	14.6	13.2
Cutting	12.1	10.9	10.2	11.2	15.1	10.4
**Rosmarinic acid (mg per 100 g) [1]**						
Propagule	*Weeks after planting*					
	3	6	9	11	13	15
Seed	42.1 bCD	46.9 aBCD	24.6 aD	158.7 aA	78.4 bBC	93.7 bB
Cutting	108.8 aB	35.7 aC	31.1 aC	162.3 aA	137.2 aAB	131.6 aAB
**Luteolin (mg per 100 g)#**						
Propagule	*Weeks after planting*					
	3	6	9	11	13	15
Seed	4.1	2.9	3.3	4.2	2.6	8.3 aA
Cutting	1.1	3.5	5.1	4.0	6.2	11.2 aA
**Apigenin (mg per 100 g)#**						
Propagule	*Weeks after planting*					
	3	6	9	11	13	15
Seed	2.2	1.5	1.5	1.8	1.5	4.9
Cutting	1.4	5.7	1.6	1.4	5.7	5.6

[1]Means followed by the same letter do not differ statistically according to the Tukey test at the 5% probability level. Lowercase letters indicate differences between methods of propagation within the same harvest date (column) and uppercase letters indicate differences among different dates of harvesting within the same propagation method (row). #Interaction effect not statistically significant.

Table S9. Analysis of variance (ANOVA) table for contents of luteolin glucoside (LuGLS), apigenin glucoside (ApGLS), luteolin glucuronide (LuGLR), apigenin glucuronide (ApGLR), luteolin diglucuronide (LuGD) and apigenin diglucuronide (ApDG) in catnip plants propagated by different methods and harvested at different times within the growing season. Pittstown, State of New Jersey, United States.

Source of variation	DF	Sum of squares					
		LuGLS	ApGLS	LuGLR	ApGLR	LuDG	ApDG
Blocks	2	1765.9^{ns}	49.9^{ns}	697.9^{ns}	23.6^{ns}	737.5^{ns}	7.6^{ns}
Propagule (p)	1	35774.5*	31.1^{ns}	2534.7^{ns}	17.7^{ns}	24123.8*	0.8^{ns}
Whole-Plot error	2	912.4	30.7	332.3	4.8	733.1	8.2
Weeks (w)	5	101656.6**	4979.5**	57980.2**	2849.8**	90265.9**	2827.5**
Interaction p×w	5	35824.1**	2691.8**	54592.7**	1182.6**	17995.3**	675.6**
Split-Plot Error	20	4647.7	387.2	3502.0	94.6	3055.3	119.6
Total	35	180581.4	8170.3	119639.8	4173.3	136910.9	3639.5

* Statistically significant ($p \leq 0.05$); ** Statistically significant ($p \leq 0.01$); ^{ns} Not statistically different ($p > 0.05$); DF: degrees of freedom.

Table S10. Analysis of variance (ANOVA) table for contents of luteolin glucoside (LuGLS), apigenin glucoside (ApGLS), luteolin glucuronide (LuGLR), apigenin glucuronide (ApGLR), luteolin diglucuronide (LuGD) and apigenin diglucuronide (ApDG) in catnip plants propagated by different methods and harvested at different times within the growing season. Upper Deerfield, State of New Jersey, United States.

Source of variation	DF	Sum of squares					
		LuGLS	ApGLS	LuGLR	ApGLR	LuDG	ApDG
Blocks	2	2297.9^{ns}	41.6^{ns}	370.8^{ns}	6.2^{ns}	549.8^{ns}	22.1^{ns}
Propagule (p)	1	156.4^{ns}	26.6^{ns}	1457.3^{ns}	18.6^{ns}	272.9^{ns}	21.3^{ns}
Whole-Plot error	2	1826.5	146.3	1729.2	41.5	1196.2	24.3
Weeks (w)	5	135872.2**	1355.6**	53069.9**	1582.5**	103053.4**	723.8**
Interaction p×w	5	19330.5**	424.6*	33474.4**	1055.1**	20875.3**	510.4**
Split-Plot Error	20	17095.7	492.8	6479.8	168.6	5361.1	355.8
Total	35	176579.3	2487.6	96581.7	2872.6	131308.7	1657.7

* Statistically significant ($p \leq 0.05$); ** Statistically significant ($p \leq 0.01$); ^{ns} Not statistically different ($p > 0.05$); DF: degrees of freedom.

molecules

MDPI

Table S11. Analysis of variance (ANOVA) table for contents of luteolin glucoside, apigenin glucoside, luteolin glucuronide, apigenin glucuronide, luteolin diglucuronide and apigenin diglucuronide in catnip plants propagated by different methods and harvested at different times within the growing season. Pittstown, State of New Jersey, United States.

**Luteolin glucoside (mg per 100 g)** [1]						
Propagule	*Weeks after planting*					
	3	6	9	11	13	15
Seed	129.4 aC	285.1 aA	257.9 aA	195.4 aB	179.7 aB	73.5 bD
Cutting	121.0 aB	233.1 bA	92.4 bB	94.9 bB	98.6 bB	102.8 aB
**Apigenin glucoside (mg per 100 g)**						
Propagule	*Weeks after planting*					
	3	6	9	11	13	15
Seed	4.5 aBC	7.7 bBC	38.8 aA	38.9 aA	14.2 aB	2.6 aC
Cutting	5.1 aB	39.3 aA	14.0 bB	29.6 bA	3.4 bB	4.2 aB
**Luteolin glucuronide (g per plant)**						
Propagule	*Weeks after planting*					
	3	6	9	11	13	15
Seed	27.7 aB	57.9 bB	160.1 aA	142.9 aA	143.7 aA	29.4 aB
Cutting	27.0 aD	187.1 aA	72.5 bBC	100.5 bB	34.9 bD	38.9 aCD
**Apigenin glucuronide (mg per plant)**						
Propagule	*Weeks after planting*					
	3	6	9	11	13	15
Seed	3.8 aC	18.3 bB	28.8 aA	27.6 aA	16.3 aB	6.0 aC
Cutting	6.5 aC	38.7 aA	14.9 bB	19.2 bB	5.8 bC	7.4 aC
**Luteolin diglucuronide (g per plant)**[1]						
Propagule	*Weeks after planting*					
	3	6	9	11	13	15
Seed	130.8 aC	231.6 aA	184.9 aB	91.9 aD	55.6 aE	48.9 aE
Cutting	104.2 bB	146.3 bA	50.7 bC	49.4 bC	41.76 aC	40.8 aC
**Apigenin diglucuronide (mg per plant)**						
Propagule	*Weeks after planting*					
	3	6	9	11	13	15
Seed	12.6 bC	27.3 bA	30.9 aA	20.5 aB	13.7 aC	8.6 aC
Cutting	16.8 aB	42.0 aA	17.6 bB	19.4 aB	7.7 bC	8.4 aC

[1]Means followed by the same letter do not differ statistically according to the Tukey test at the 5% probability level. Lowercase letters indicate differences between methods of propagation within the same harvest date (column) and uppercase letters indicate differences among different dates of harvesting within the same propagation method (row).

molecules

MDPI

Table S12. Analysis of variance (ANOVA) table for contents of luteolin glucoside, apigenin glucoside, luteolin glucuronide, apigenin glucuronide, luteolin diglucuronide and apigenin diglucuronide in catnip plants propagated by different methods and harvested at different times within the growing season. Upper Deerfield, State of New Jersey, United States.

**Luteolin glucoside (mg per 100 g)**[1]						
Propagule	*Weeks after planting*					
	3	6	9	11	13	15
Seed	242.2 aA	88.9 bB	113.9 aB	265.4 aA	266.7 aA	127.2 aB
Cutting	213.8 aAB	166.4 aB	148.6 aBC	209.4 bAB	255.1 aA	85.7 aC
**Apigenin glucoside (mg per 100 g)**						
Propagule	*Weeks after planting*					
	3	6	9	11	13	15
Seed	20.4 aA	6.8 aB	5.8 aB	21.8 aA	9.9 aAB	3.7 aB
Cutting	3.6 bB	5.4 aB	7.6 aB	24.0 aA	12.0 aAB	5.5 aB
**Luteolin glucuronide (mg per plant)**						
Propagule	*Weeks after planting*					
	3	6	9	11	13	15
Seed	205.3 aA	32.5 aC	44.1 aC	119.9 aB	49.8 bC	25.4 aC
Cutting	60.3 bBC	40.3 aC	55.6 aBC	111.5 aA	91.5 aAB	41.4 aC
**Apigenin glucuronide (mg per plant)**						
Propagule	*Weeks after planting*					
	3	6	9	11	13	15
Seed	41.3 aA	10.9 aC	14.9 aBC	22.3 aB	12.2 bC	9.4 aC
Cutting	16.1 bBC	14.2 aBC	17.7 aB	25.8 aA	19.2 aAB	9.2 aC
**Luteolin diglucuronide (g per plant)**[1]						
Propagule	*Weeks after planting*					
	3	6	9	11	13	15
Seed	254.7 aA	106.2 bC	101.3 bC	111.9 aC	163.6 aB	56.3 aD
Cutting	203.3 bA	159.5 aB	155.8 aBC	115.5 aCD	91.6 bD	35.1 aE
**Apigenin diglucuronide (mg per plant)**						
Propagule	*Weeks after planting*					
	3	6	9	11	13	15
Seed	32.4 aA	12.7 aB	17.5 aB	17.5 bB	13.1 bB	12.6 aB
Cutting	19.2 bAB	18.8 aAB	19.7 aAB	25.8 aA	21.0 aAB	10.6 aB

[1]Means followed by the same letter do not differ statistically according to the Tukey test at the 5% probability level. Lowercase letters indicate differences between methods of propagation within the same harvest date (column) and uppercase letters indicate differences among different dates of harvesting within the same propagation method (row).

molecules

MDPI

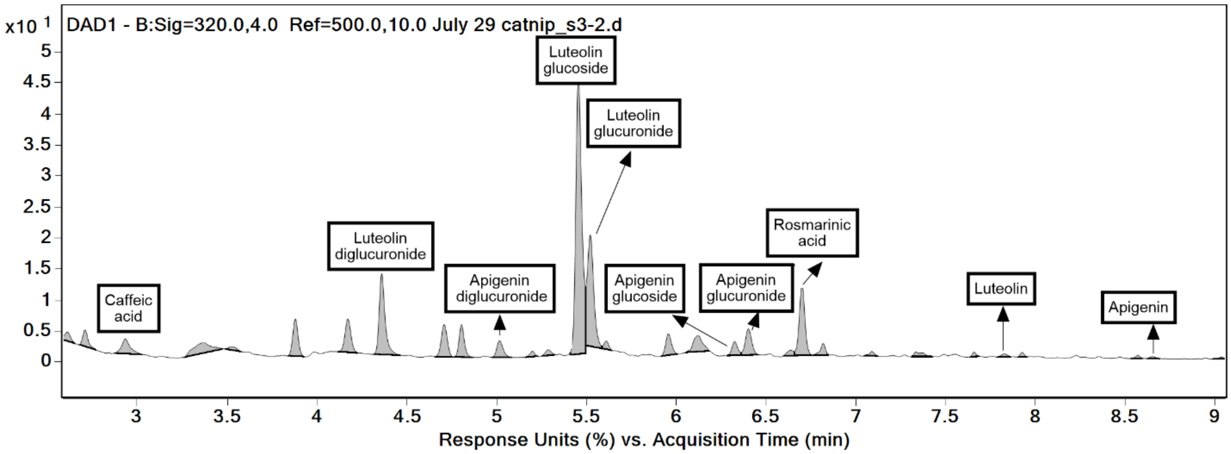

Figure S1. Representative UHPLC-DAD chromatogram (320 nm) of phenolic compounds in the methanolic extracts of *Nepeta cataria* L. aerial parts.

molecules

MDPI

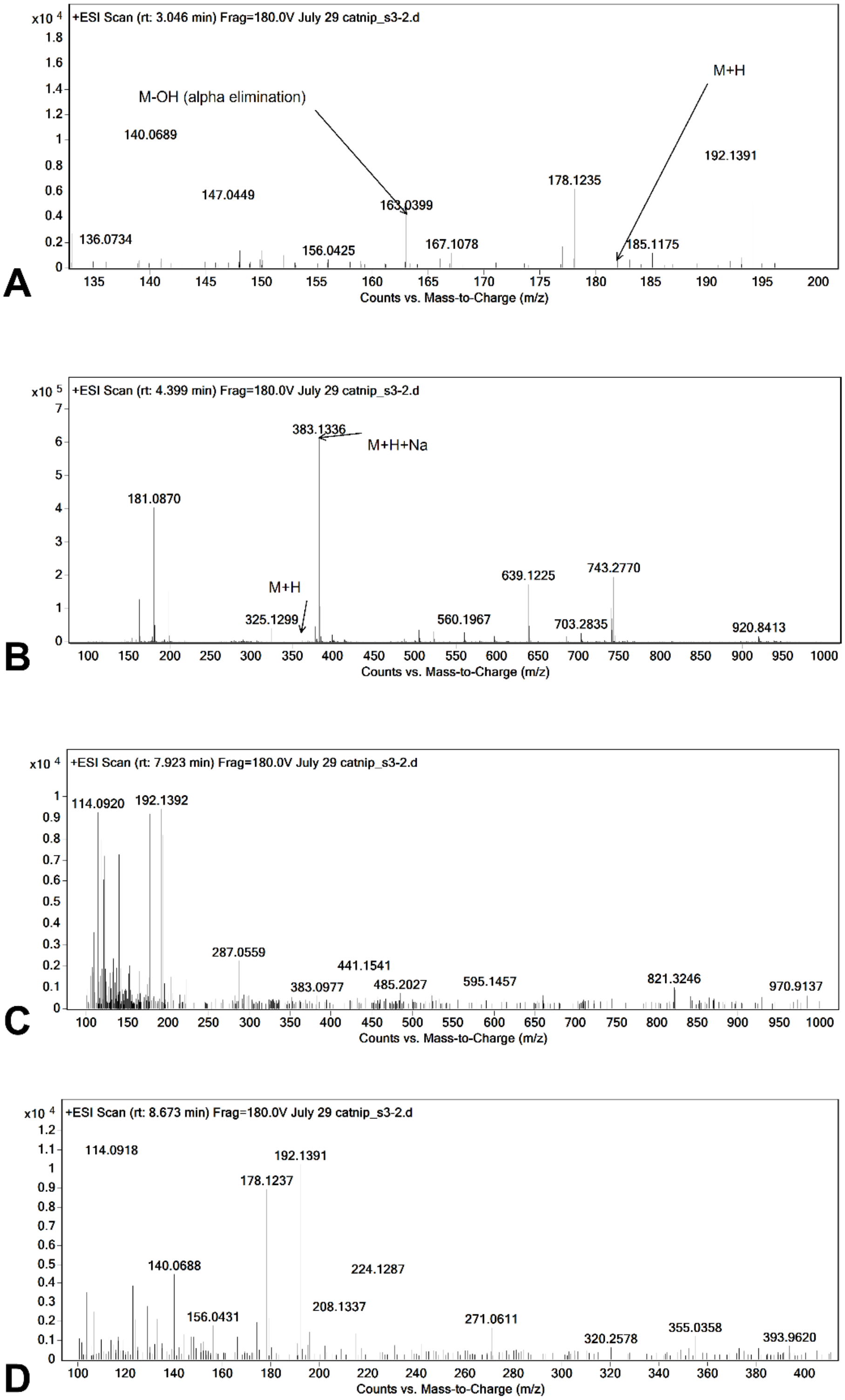

Figure S2: Mass spectra of caffeic acid (A), rosmarinic acid (B), luteolin (C), and apigenin (D) in methanolic extracts of *Nepeta cataria* L. aerial parts.

molecules

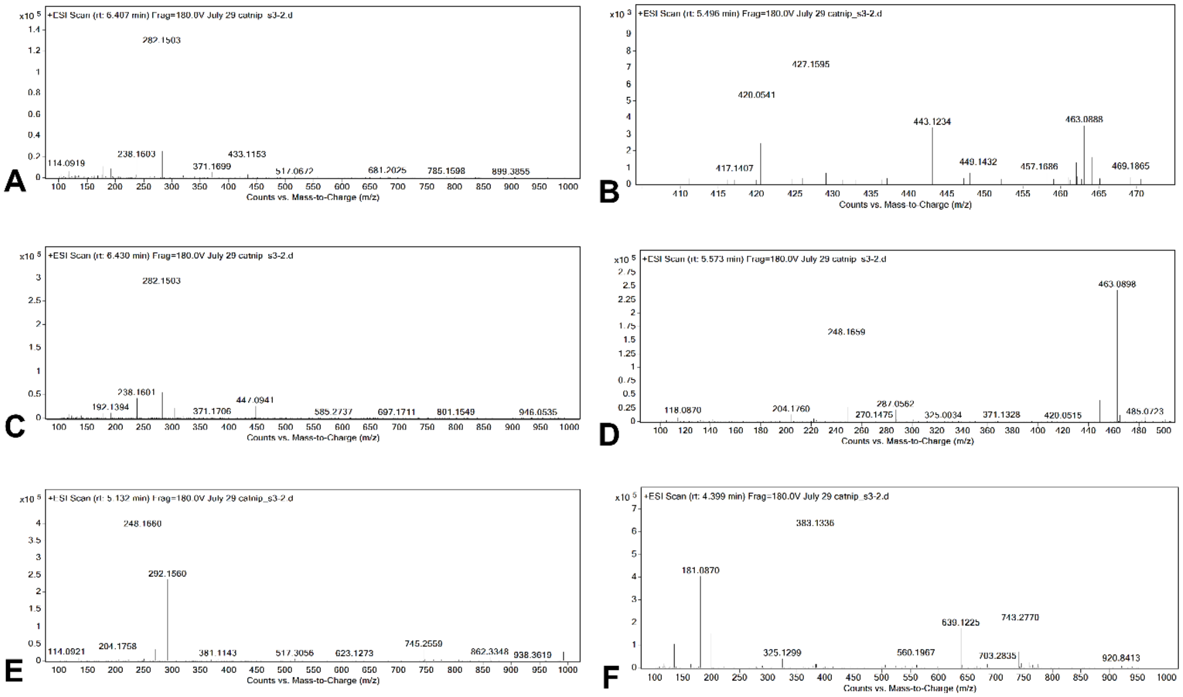

Figure S3: Mass spectra of apigenin glucoside (A), luteolin glucoside (B), apigenin glucuronide (C), luteolin glucuronide (D), apigenin diglucuronide (E), and luteolin diglucuronide (F) in methanolic extracts of *Nepeta cataria* L. aerial parts.

molecules

MDPI

Table S13. Results of Brown-Forsythe (homogeneity of variances) and Kolmogorov-Smirnov (normal distribution of residuals) tests on original and transformed data of different variables of catnip plants propagated by different methods and harvested at different times within the growing season. Pittstown, State of New Jersey, United States.

Pittstown						
	**Brown– Forsythe test**[1]			**Kolmogorov-Smirnov test**[2]		
**Variables**	Original data	Transformed to *Y=Log2(Y)*	Transformed to *Y=sin(Y)*	Original data	Transformed to *Y=Log2(Y)*	Transformed to *Y=sin(Y)*
Z,E-nepetalactone	ns	ns	ns	ns	n.a.	n.a.
E,Z-nepetalactone	ns	ns	ns	ns	n.a.	n.a.
Total nepetalactone	ns	ns	ns	ns	n.a.	n.a.
Biomass	ns	ns	ns	ns	n.a.	n.a.
Total nepetalactone yield	ns	ns	ns	*	ns	n.a.
Nepetalic acid	ns	ns	ns	ns	n.a.	n.a.
Dihydronepetalactone	ns	ns	ns	ns	n.a.	n.a.
Nepetalactam	ns	ns	ns	***	ns	n.a.
Caffeic acid	ns	ns	ns	ns	n.a.	n.a.
Rosmarinic acid	ns	ns	ns	*	ns	n.a.
Apigenin	ns	ns	ns	***	***	ns
Luteolin	ns	ns	ns	ns	n.a.	n.a.
Apigenin glucoside	ns	ns	ns	***	***	ns
Luteolin glucoside	ns	ns	ns	**	ns	n.a.
Apigenin glucuronide	ns	ns	ns	**	***	ns
Luteolin glucuronide	ns	ns	ns	ns	n.a.	n.a.
Apigenin diglucuronide	ns	ns	ns	ns	n.a.	n.a.
Luteolin diglucuronide	ns	ns	ns	***	ns	n.a.

[1]Testing the null hypothesis that the variances of the populations are homogenous. [2]Testing the null hypothesis that the residuals are normally distributed. ns: not significant, the null hypothesis was not rejected. *Null hypothesis rejected at the 5% probability level. **Null hypothesis rejected at 1% probability level ***Null hypothesis rejected at 0.1% probability level. n.a.: not applicable.

molecules

MDPI

Table S14. Results of Brown-Forsythe (homogeneity of variances) and Kolmogorov-Smirnov (normal distribution of residuals) tests on original and transformed data of different variables of catnip plants propagated by different methods and harvested at different times within the growing season. Upper Deerfield, State of New Jersey, United States.

Upper Deerfield						
	**Brown– Forsythe test[1]**			**Kolmogorov-Smirnov test[2]**		
**Variables**	Original data	Transformed to *Y=Log2(Y)*	Transformed to *Y=sin(Y)*	Original data	Transformed to *Y=Log2(Y)*	Transformed to *Y=sin(Y)*
Z,E-nepetalactone	ns	ns	ns	*	ns	n.a.
E,Z-nepetalactone	ns	ns	ns	ns	n.a.	n.a.
Total nepetalactone	ns	ns	ns	ns	n.a.	n.a.
Biomass	ns	ns	ns	ns	n.a.	n.a.
Total nepetalactone yield	ns	ns	ns	*	ns	n.a
Nepetalic acid	ns	ns	ns	ns	n.a.	n.a.
Dihydronepetalactone	ns	ns	ns	ns	n.a.	n.a.
Nepetalactam	ns	ns	ns	*	ns	n.a.
Caffeic acid	ns	ns	ns	*	ns	n.a.
Rosmarinic acid	ns	ns	ns	*	ns	n.a.
Apigenin	ns	ns	ns	ns	n.a.	n.a.
Luteolin	ns	ns	ns	*	ns	n.a.
Apigenin glucoside	ns	ns	ns	**	**	ns
Luteolin glucoside	ns	ns	ns	*	ns	n.a.
Apigenin glucuronide	ns	ns	ns	**	ns	n.a.
Luteolin glucuronide	ns	ns	ns	ns	n.a.	n.a.
Apigenin diglucuronide	ns	ns	ns	***	***	ns
Luteolin diglucuronide	ns	ns	ns	ns	n.a.	n.a.

[1]Testing the null hypothesis that the variances of the populations are homogenous. [2]Testing the null hypothesis that the residuals are normally distributed. ns: not significant, the null hypothesis was not rejected. *Null hypothesis rejected at the 5% probability level. **Null hypothesis rejected at 1% probability level ***Null hypothesis rejected at 0.1% probability level. n.a.: not applicable.
